# Development of a 96-Well Electrophilic Allergen Screening Assay for Skin Sensitization Using a Measurement Science Approach

**DOI:** 10.3390/toxics10050257

**Published:** 2022-05-17

**Authors:** Elijah J. Petersen, Richard Uhl, Blaza Toman, John T. Elliott, Judy Strickland, James Truax, John Gordon

**Affiliations:** 1 Biosystems and Biomaterials Division, Material Measurement Laboratory, National Institute of Standards and Technology (NIST), 100 Bureau Drive, Gaithersburg, MD 20899, USA; john.elliott@nist.gov; 2 Division of Laboratory Sciences, Chemistry, US Consumer Product Safety Commission (CPSC), 5 Research Place, Rockville, MD 20850, USA; ruhl@cpsc.gov; 3 Statistical Engineering Division, Information Technology Laboratory, National Institute of Standards and Technology (NIST), 100 Bureau Drive, Gaithersburg, MD 20899, USA; blaza.toman@nist.gov; 4 Inotiv-RTP., 601 Keystone Park Drive, Suite 800, Morrisville, NC 27560, USA; judy.strickland@inotivco.com (J.S.); jim.truax@inotivco.com (J.T.); 5 Division of Toxicology and Risk Assessment, US Consumer Product Safety Commission (CPSC), 5 Research Place, Rockville, MD 20850, USA; jgordon@cpsc.gov

**Keywords:** skin sensitization, adverse outcome pathway, direct peptide reactivity assay, in vitro method, new approach methodology, measurement science, metrology

## Abstract

The Electrophilic Allergen Screening Assay (EASA) has emerged as a promising *in chemico* method to detect the first key event in the adverse outcome pathway (AOP) for skin sensitization. This assay functions by assessing the depletion of one of two probe molecules (4-nitrobenzenethiol (NBT) and pyridoxylamine (PDA)) in the presence of a test compound (TC). The initial development of EASA utilized a cuvette format resulting in multiple measurement challenges such as low throughput and the inability to include adequate control measurements. In this study, we describe the redesign of EASA into a 96-well plate format that incorporates in-process control measurements to quantify key sources of variability each time the assay is run. The data from the analysis of 67 TCs using the 96-well format had 77% concordance with animal data from the local lymph node assay (LLNA), a result consistent with that for the direct peptide reactivity assay (DPRA), an OECD test guideline (442C) protein binding assay. Overall, the measurement science approach described here provides steps during assay development that can be taken to increase confidence of *in chemico* assays by attempting to fully characterize the sources of variability and potential biases and incorporate in-process control measurements into the assay.

## 1. Introduction

Extensive research efforts have focused in recent years on the development of in vitro test methods to assess the potential for a substance, formulation, or product to cause skin toxicity (e.g., sensitization and irritation) [1,2,3,4,5,6,7,8,9,10,11,12,13,14]. One influential factor driving this focus has been new regulations in some jurisdictions (e.g., the European Union) banning animal testing for cosmetic products (2009/1223/EU), and other new regulations such as the Frank Lautenberg act supporting the use of new approach methodologies (NAMs) for testing new chemicals for regulatory review by the U.S. Environmental Protection Agency (EPA) [15]. The EPA set forth an interim policy in 2018 stating that they will recommend the use of NAMs instead of animal testing for assessing skin sensitization hazard [16], while other US agencies (e.g., the Consumer Product Safety Commission) are increasing efforts to accept NAMs for regulatory purposes. However, some countries still require animal testing for skin sensitization [17]. This poses challenges for industry as completing the requisite tests for one location may not be accepted or hinder selling products in another area. The development of robust, standardized methods for NAMs is critical to build confidence in these methods, and thus support a harmonized international regulatory framework that can facilitate commerce and reduce animal testing [18].

To predict the results from an in vivo standard skin sensitization method, NAMs have been developed for some of the key events (KEs) of the skin sensitization adverse outcome pathway (AOP) for compounds where sensitization is initiated by covalent binding to proteins [19]. There are several KEs in this AOP for chemicals that can penetrate through human skin: KE1) covalent bonding of a substance to skin proteins; KE2) activation of keratinocytes (this can be tested, for example, by the expression of genes of the antioxidant response element as part of the Keap1/Nrf2 pathway after contact with the substance); KE3) activation of dendritic cells (tested by cell surface markers present after maturation of dendritic cells following the uptake and processing of the substance); and lastly KE4) antigen presentation, activation, and proliferation of specific T-cells. Allergic contact dermatitis and epidermal inflammation occur following re-exposure to a substance due to T cell-mediated cell death. KEs 1, 2, and 3 can be tested using, for example, the direct peptide reactivity assay (DPRA) [20,21,22], the keratinocyte activation test KeratinoSens [23,24], and the human cell-line activation test (h-CLAT) [25,26], respectively. Combinations of the results of these assays have been integrated using a range of computational approaches and compared to results from the frequently used in vivo animal test for skin sensitization, the murine local lymph node assay (LLNA), and human skin sensitization data [27,28,29,30,31]. These data integration procedures have been referred to as defined approaches when they are characterized by fixed data interpretation procedures applied to specific data sources. Some defined approaches have recently gained regulatory acceptance for hazard classification [16,32].

Nevertheless, there are limitations to some of the assays that have been used to assess the protein binding step such as the DPRA [20,21], which has become an OECD test guideline [22]. For example, the DPRA is typically conducted using high performance liquid chromatography (HPLC), which limits the throughput and the number of quality control measurements that can be assessed each time the assay is performed as two probe molecules are tested (with each taking up to 54 h (24 h incubation and 30-h maximum HPLC run time)). Additional limitations to the DPRA include incompatible solubility between peptides and test chemicals and non-specific modifications of the peptides due to oxidative reactions [33,34].

Two cuvette-based electrophilic binding assays that measure the fluorescence of an amine-based probe, pyridoxylamine (PDA) or the absorbance of a thiol-based probe, 4-nitrobenzenethiol (NBT), have been previously described in detail [34,35]. Both probes were used as the NBT probe did not detect amine-based skin sensitizers and the PDA probe did not react with thiol-based skin sensitizers. In both assays, reaction rates correlated positively with LLNA EC_3_ values (the concentration that induces a stimulation index of three, the threshold positive response) and neither assay reacted with non-sensitizers. These assays evaluate the extent to which test compounds (TCs) interact with the probe molecules. The PDA and NBT assays were nominated as the “Electrophilic Allergen Screening Assay” (EASA) for validation to the Interagency Coordinating Committee on the Validation of Alternative Methods in 2012 [36]. However, the use of cuvettes limited the assay throughput and capacity to perform control measurements given that many cuvette readers can only hold up to eight samples at a time. Moreover, there is a limited availability of cuvette-based spectrophotometers in contract testing organizations when compared to plate readers. Furthermore, the use of a cuvette-based system results in an increased volume of organic waste when compared to a 96-well plate format. Thus, there is a need for a higher throughput method to assess skin sensitization that is robust and can incorporate numerous in-process control measurements to comprehensively understand the assay performance each time it is performed. This could enable the identification and correction of potential biases.

In this study, we apply a measurement science process to redesign EASA intended for use in place of the DPRA to assess KE1 of the AOP for skin sensitization. The new design uses a plate reader with several in-process control measurements incorporated into the 96-well plate layout. In this assay, NBT and PDA mimic the binding reactions with thiol or amine reactive sensitizers and represent cysteine and lysine side chains, respectively [34,35]. Extensive robustness testing was conducted to comprehensively understand sources of variability in the assay. The approach used in this assay with cause-and-effect (C&E) analyses and robustness testing provides a model for the development of robust NAMs. Ninety-two TCs (Table 1) were tested in this assay to evaluate its use across a range of chemicals and to uncover potential biases. These results were then compared to LLNA results to understand the predictive ability of this assay.

## 2. Materials and Methods

**Figure 1:**
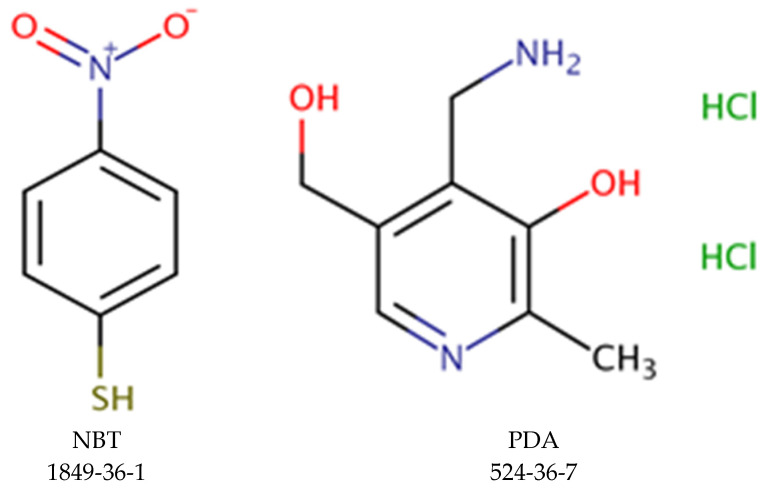
Chemical structure of probe molecules.

Two different probe molecules were tested in this assay: NBT and PDA (Figure 1).

**Figure 2:**
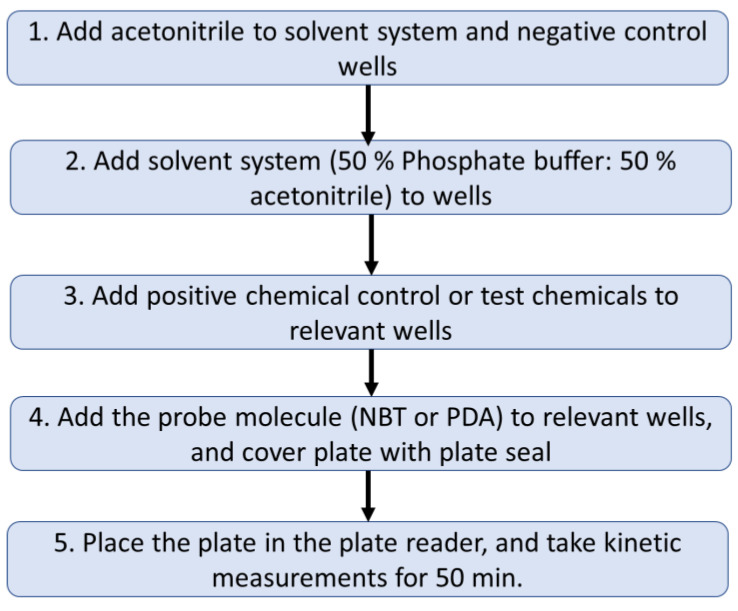
Flow chart outlining the main steps for the EASA method.

To test binding to PDA, protocols were developed based upon changes in absorbance or fluorescence readings, while the NBT assay only monitored changes in absorbance, as NBT did not have a characteristic fluorescence signal. Unlike the original EASA methods, which were based on a single cuvette format [34,35], this revised EASA method used a plate reader and 96-well microplates to enable the incorporation of several in-process control measurements and increase the number of compounds tested per assay performed. During this assay, reaction of a *TC* with NBT or PDA caused a decrease in the absorbance or fluorescence signal. A flowchart of this assay is provided in Figure 2.

The three assays (NBT absorbance, PDA absorbance and PDA fluorescence) that comprise the EASA assay, were conducted with similar procedures with the exception of the positive control (PC) chemicals and probe molecule concentrations. The solvent system (SS) was made using a 50:50 mixture of phosphate buffer (PB) (0.1 mol/L, pH 7.4 ± 0.2) and acetonitrile. All three assays used 1 mmol/L probe (NBT or PDA) stock solutions. Probe solutions were bath sonicated for approximately 1 min to 2 min to aid dissolution. The NBT solution was stable when stored up to 7 d at −20 ± 2 °C and protected from light (storing the solution in amber vials wrapped in aluminum foil is recommended). The NBT stock solution was aliquoted into smaller volumes for daily use to avoid repeated freezing and thawing. The stock solutions were diluted to make working probe solutions for all three assays as follows. For the NBT and PDA absorbance assays a 1:8 (0.125 mmol/L) working probe solution using SS was made. For the PDA fluorescence assay, a 1:120 (0.008 mmol/L) working probe solution was made in SS. To protect the NBT working solution from light, the solution was stored in amber vials that were wrapped in aluminum foil, and only red light was used in the laboratory when preparing the NBT working solution. Finally, the PCs for all three assays were prepared: 3 mmol/L benzyl bromide in acetonitrile for the NBT absorbance assay; 1 mmol/L glutaraldehyde in acetonitrile for the PDA absorbance assay; and lastly, 0.1 mmol/L glutaraldehyde in acetonitrile for the PDA fluorescence assay. TCs were prepared at 10 mmol/L in acetonitrile and tested at 2 mmol/L (40 µL *TC* with 160 µL working probe) concentrations. The assay was run at room temperature (21 ± 2 °C).

**Figure 3:**
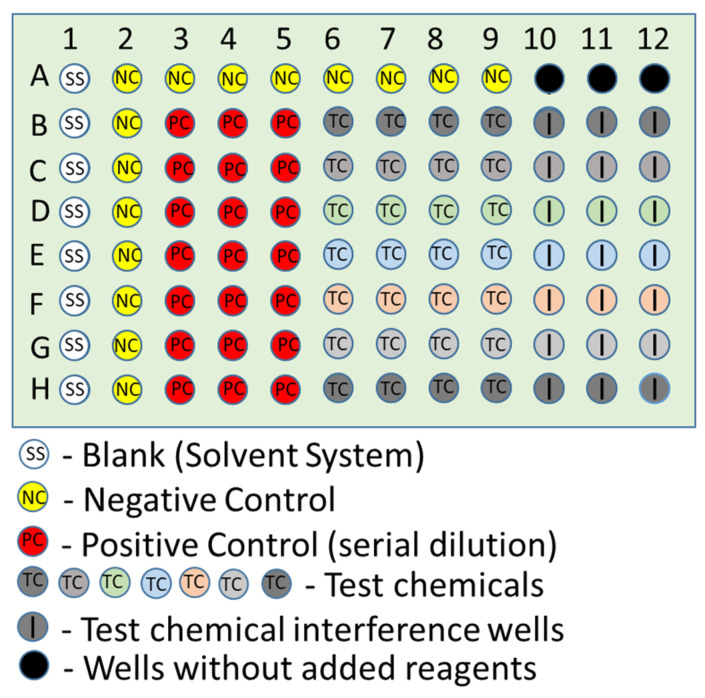
96 well plate design.

The plate layout for all three assays was identical (Figure 3). First, 40 µL of acetonitrile was added to column 1 of the 96-well plate, which serves as the plate blank for the negative and PCs. Next, 40 µL acetonitrile was added to Column 2 and Row A (columns 2 through 9). These wells served as the negative controls (NC) for the assay (after the addition of the probe). The *PC* consisted of seven triplicate 1:2 serial dilutions starting with 40 µL of the *PC* stock solution in Columns 3 to 5, Row B, with the serial dilution ending in Columns 3 to 5, Row H. The final volume of the added *PC* in each well was 40 µL. The TCs were then loaded to columns 6 to 12 horizontally starting in Row B (one *TC* per row). Next, 160 µL of SS was added to columns 1, 10, 11, and 12. When performing the NBT absorbance assay, the red light was turned on and all laboratory lights turned off before adding the NBT working solution. Next, 160 µL of the appropriate working probe solution was immediately added to columns 2 through 9 vertically using a multi-channel pipette, adding vertically from left to right. The working probe solution was not added to columns 1, 10, 11, or 12. The total volume for each well was 200 µL. The working probe solution for all three assays was added last, and the timer for incubation was started when the first pipetting step of the probe solution was performed. The plates were examined for visually observable bubbles, which were then removed if possible using a 10 µL pipette tip. A VIEWseal plate seal was placed on the plate. Absorbance or fluorescence data were recorded at approximately 5 min ± 30 s, 20 ± 2 min, 35 ± 2 min, and 50 ± 2 min.

The NBT absorbance assay is monitored at 412 nm absorbance. The PDA absorbance assay is monitored at 324 nm, and the PDA fluorescence assay is monitored at an excitation wavelength of 324 nm and an emission wavelength of 398 nm.

If the *TC* does not go into solution using acetonitrile, or if during the performance of the assay there is too much interference from the *TC* or if precipitation is observed, it is possible to test the *TC* using SS or PB as the solvent instead. When using SS or PB as the solvent, substitute all solutions for the blank NCs and PCs with the same *TC* solubilizing buffer, except for the benzyl bromide, which should be dissolved in acetonitrile. All other controls and solutions are made at the same concentrations previously discussed in this section for both the NBT absorbance and PDA absorbance assays. When performing the PDA fluorescence assay using PB as the solvent, the *PC* stock solution was 0.05 mmol/L glutaraldehyde, and the stock and working PDA probe solutions were changed to 0.3 mmol/L and 2.4 µmol/L in PB, respectively.

The PDA and NBT concentrations used in this study (0.1 mmol/L for the NBT and PDA absorbance measurements and 0.0064 mmol/L for the PDA fluorescence measurements) are higher than those used in the previous studies (0.05 mmol/L, 0.01 mmol/L and 0.005 mmol/L for the NBT, PDA absorbance, and PDA fluorescence assays, respectively) [34,35]. The different concentrations were chosen to maximize the amount of signal and dynamic range for the *NC* wells relative to the readings for the background, namely the SS wells. The stock concentration of the *TC* was 10 mmol/L in both studies, but there was a higher amount of dilution in this study (1:5) compared to the previous studies (1:2) [34,35]. The concentration in this study was chosen to balance the potential for detecting reactivity of the *TC* and for the potential of interference from the *TC* in the absorbance and fluorescence measurements as well as solubility issues.

It is important to note the potential safety risks involved in handling skin sensitizers. Therefore, a safe operating procedure is provided in the Supplementary Materials (Electrophilic Allergen Screening Assay (EASA) Safe Operating Procedure).

### 2.1. Materials

The plate sealing tape used in this study was the Greiner Bio-One Viewseal adhesive plate sealer, which is resistant to acetonitrile degradation. The 96-well plates used were the Greiner Bio-One UV-Star 96-well clear bottom UV spectroscopy microplates composed of cycloolefin, which are also designed to be resistant to acetonitrile degradation. Thermo Scientific microplate sealing tape was also tested. Polystyrene reservoirs for multi-channel pipettes from Fisher Scientific were used, as was an Eppendorf multi-channel pipette and Eppendorf repeater pipette. A red bulb (25 W, A19 type bulb, GE Lighting 49727) was used to provide lighting during preparation and addition of the probe solution during the NBT assay as red light was determined to have a significantly lower capacity than white light to cause photodegradation of NBT. Phosphate buffer (1 mol/L, pH 7.4), acetonitrile (reagent grade, 98%), PDA (analytical standard, ≥ 98%), NBT (technical grade, 80%), and benzyl bromide (reagent grade, 98%) were all obtained from Sigma-Aldrich. Glutaraldehyde (50% certified) was obtained from Fisher Scientific. NBT was also purchased from Matrix Scientific (Columbia, SC, USA) and tested for comparison. Unless stated otherwise, plates were evaluated using a Synergy MX Biotek plate reader.

### 2.2. Robustness Testing

Based on the C&E analysis, the following measurements were made to investigate potential sources of variability in the assay through the following robustness testing: (1) plate reader performance was evaluated by the homogeneity and potential for edge effects (i.e., different results for cells along the edge of the plate) by adding either a *NC* (probe molecule dissolved in SS) or *PC* to the entire 96-well plate using a multichannel pipette, and by assessing the linearity of the plate reader; (2) the impact of plate selection was evaluated by testing polystyrene or cycloolefin plates for up to 120 min after the addition of SS (50:50 ratio of acetonitrile: phosphate buffer (0.1 mol/L, pH 7.4)); (3) cross-talk was evaluated by adding PDA to only row E of a plate while the other rows had SS added and evaluating if there was increased fluorescence signal in the adjacent rows at the PDA wavelength; (4) the stability of Thermo Scientific microplate sealing tape and ViewSeal plate seal were evaluated for up to 120 min after the addition of SS; (5) performing the assay for durations up to 120 min; (6) potential for condensation to be observed during the assay duration on the plate seal using two plate readers (Synergy MX Biotek or Tecan Spark 20) and different instrument settings (i.e., the cooling system being turned off or on); (7) reservoirs composed of polystyrene or polypropylene were evaluated after the addition of acetonitrile or SS; (8) the impact of the pipetting direction to add the *NC* to the plate (top to bottom versus left to right); (9) the impact of bias from TCs that absorb or fluoresce at wavelengths similar to the probe molecules; (10) the impact of different solvents; (11) different potential PCs; (12) different initial *TC* concentration (2 mmol/L, 0.2 mmol/L, or 0.02 mmol/L); (13) potential photodegradation of the probe molecule while preparing the plate, (14) different batches and manufacturers of the probe molecules, and (15) the repeatability of the in-process control measurements across time.

### 2.3. Evaluation of Probe Depletion

Ninety-two chemicals were tested (see Table 1 for a list of the TCs and Table S1 for the suppliers of the TCs). Sixty of the TCs were selected based on a National Toxicology Program (NTP) study on the performance of in vitro assays for skin sensitization while the remaining TCs were selected from those evaluated in [37] with both DPRA and LLNA data. The putative reaction mechanism for each chemical was determined using OECD QSAR Toolbox version 4.3; the results are provided in Table S1.

The percentage depletion was calculated for absorbance and fluorescence measurements using the following equations for *NC* or *PC* wells or TCs:

(1) $$NC,\;PC\;well\;\%\;depletion=\left(1-\frac{NC,\;\;PC\;well-\bar{blank}}{\bar{NC}-\bar{blank}}\right)\times 100\%$$

(2) $$TC\;\%\;Depletion=\left(1-\frac{\bar{TC}-\bar{TC_{blank}}}{\bar{NC}-\bar{blank}}\right)\times 100\%$$

where $\bar{blank}$ indicates the mean of the SS wells, $\bar{NC}$ indicates the mean of the *NC* wells, $\bar{TC\;}$ indicates the mean of the *TC* wells for each compound (note that these wells also contain the probe molecule), and $\bar{TC_{blank}}$ indicates the mean of the *TC* blank wells for each compound (note that these wells do not contain the probe molecule). The overscore indicates a mean value, while the values without an overscore indicate individual wells.

In addition, the absolute depletion value (in absorbance or fluorescence units) was also calculated for each compound.

(3) $$Depletion=\left(\bar{NC}-\bar{blank}\right)-\left(\bar{TC}-\bar{TC_{blank}}\right)$$

Equation (2) can be rewritten as follows to show the relationship between depletion and percentage depletion:

(4) $$TC\;\%\;depletion=\left(\frac{Depletion}{\bar{NC}-\bar{blank}}\right)\times 100\%$$

Seven TCs (tri-n-octylphospine oxide, dicyclohexylcarbodiimide, triethanolamine, pentaerythritol triacrylate, clarithromycin, 5-amino-o-cresol, and o-benzyl-p-chlorophenol) showed results that were close to the threshold for determining if the substance caused a detectable amount of depletion of the probe molecules. To assess the uncertainty in the test results for these “borderline” compounds, they were tested 3 or 4 times on separate days with new *TC* stock solutions prepared each day.

### 2.4. Statistical Analysis

Depletion of the probe molecule indicates whether a substance is a binder or nonbinder. Results were evaluated using three different approaches to assess if there was a significant depletion of the probe molecule: (1) by comparing the *TC* percentage depletion to a factor times the standard deviation of the NC; (2) by performing a statistical calculation using a frequentist approach and t-statistics; and (3) by performing a statistical calculation using a Markov Chain Monte Carlo Bayesian analysis.

If the distributions of *NC*, *blank*, *TC*, and *TC_blank_* are assumed to be Gaussian, then the averages are also Gaussian. Each factor in Equations (3) and (4) (blank, *NC*, *TC blank*, and *TC*) was used to evaluate the uncertainty in the assay. The standard deviation of $\left(\bar{NC}-\bar{blank}\right)-\left(\bar{TC}-\bar{TC_{blank}}\right)$ is $\sqrt{\frac{s_{NC}^{2}}{15}+\frac{s_{blank}^{2}}{8}+\frac{s_{TC}^{2}}{4}+\frac{s_{TC_{blank}}^{2}}{3}}$ where s is the standard deviation and *s_NC_*, for example, is the standard deviation for the *NC*. The denominators in the fractions are based on the number of replicate wells (i.e., n − 1). There are 15 *NC* replicates, 8 blank replicates, 4 *TC* replicates and 3 *TC* blank replicates. The statistic $T=\frac{\left(\bar{NC}-\bar{blank}\right)-\left(\bar{TC}-\bar{TC_{blank}}\right)}{\sqrt{\frac{s_{NC}^{2}}{15}+\frac{s_{blank}^{2}}{8}+\frac{s_{TC}^{2}}{4}+\frac{s_{TC_{blank}}^{2}}{3}}}$ has a Student t distribution with degrees of freedom estimated by the Welsh–Satterthwaite formula:

(5) $$df=\frac{{\left(\frac{s_{NC}^{2}}{15}+\frac{s_{blank}^{2}}{8}+\frac{s_{TC}^{2}}{4}+\frac{s_{TC_{blank}}^{2}}{3}\right)}^{2}}{\frac{{\left(\frac{s_{NC}^{2}}{15}\right)}^{2}}{14}+\frac{{\left(\frac{s_{blank}^{2}}{8}\right)}^{2}}{7}+\frac{{\left(\frac{s_{TC}^{2}}{4}\right)}^{2}}{3}+\frac{{\left(\frac{s_{TC_{blank}}^{2}}{3}\right)}^{2}}{2}}$$

Based on this information, it is possible to calculate *p* values for a given chemical based on the *T* and *df* values. By comparing the *p* value to a specified value of α, it is determined whether a chemical is a binder with this degree of statistical confidence. The value of α is the type I error (the probability that the *TC* will be labeled as a binder when it is a nonbinder). The type II error (the probability that the *TC* will be labeled as a nonbinder when it is a binder) rate is also of interest, and is calculated under the alternative hypothesis, that is, “depletion is not equal to 0”. Since this is not a simple hypothesis, the type II error rate needs to be calculated under various alternatives by assuming that T is equal to some constant other than 0 (e.g., 1, 2, 3, 4). Based on the α selected, the type II error rate can be calculated for these various alternative hypotheses. It is not possible to lower both the type I and type II error rates simultaneously. If the type II error rate for a given α is too high, the α needs to be increased. Calculator worksheets included in the Supplementary Materials function as the prediction model and perform the frequentist analysis after pasting in data from a particular run and information about specifications for the different in-process control measurements. Calculators are included for both the NBT and PDA fluorescence assay as well as a completed version of the calculator using data from an NBT run.

In addition to the above approach, we tried an alternative analysis based on a Bayesian model [51,52,53], analyzed using Markov Chain Monte Carlo programmed in OpenBUGS [54]. The advantage of this approach is its ability to do exact calculations of uncertainty without resorting to the Welsh–Satterthwaite approximation. As above, we assumed that all measurements were Gaussian. We used the usual Gaussian prior distributions for all the means, and Gamma distributions for all the unknown variances [52]. We calculated *Depletion* (Equation (3)) for each TC. If the lower bound of the confidence interval was greater than 0, the *TC* was labeled as a binder. Otherwise, it was labeled as a nonbinder.

The quality of the absorbance and fluorescence assays were directly compared using *Z-factors* [55], a term that provides a dimensionless value that represents discrimination ability by taking into account the dynamic range of the assay and its variability. *Z-factors* range from a maximum of 1 for excellent assays, while those near 0 indicate that the assay can only provide a “yes” or “no” response. *Z-factors* were calculated using the following equation [55]:

(6) $$Z-factor=1-\frac{3s_{sample}+3s_{PC}}{\bar{NC}-\bar{blank}}$$

where *s_sample_* refers to the average standard deviation of the test samples after the removal of compounds with percent depletion values less than 5%, and the standard deviation of TCs that are three times greater than the average of *s_sample_* after the removal of those compounds (1, 4, and 4 TCs were removed for the NBT, PDA absorbance, and PDA fluorescence assays, respectively); compounds with high *s_sample_* values were removed as some compounds with high background interference led to *s_sample_* values much higher than those for the other compounds. *s_PC_* refers to the standard deviation for the PC, glutaraldehyde for the PDA assays tested at 2 mmol/L, or tetraethylthiuramdisulfide at 2 mmol/L for the NBT assay (the compound with the highest percentage depletion for this assay), all of which yielded percentage depletion values of ≈99%, the maximum amount of depletion observed in the assay.

To evaluate if there was cross-talk among rows, analysis of variance (ANOVA) was performed among the rows without PDA using GraphPad Prism (version 5.0). GraphPad Prism was also used to fit a linear regression among the results from the different pipetting steps for the *NC* wells, and evaluate if the slope was significantly different than 0 (α = 0.05).

The data to investigate the impact of pipetting order (top to bottom versus left to right) was evaluated based on a Gaussian random effects model for the measurement in each of the wells (mean plus a random plate effect) [51]. It was estimated using Bayesian Markov Chain Monte Carlo Simulation with noninformative priors. The effects of the pipetting order were determined by evaluating if the 95% confidence intervals overlapped.

If either the NBT absorbance, PDA absorbance, or PDA fluorescence assay indicated statistically significant depletion, that run would indicate that the *TC* is a binder. Otherwise, that run would indicate that the *TC* is a nonbinder. For TCs that were evaluated multiple times, they were labeled as binders or nonbinders, depending upon which outcome occurred in a majority of runs; each run includes analysis of the *TC* using all three assays. TCs were labeled as “inconclusive” if separate runs yielded binder and nonbinder an equal number of times.

For the 44 TCs with both EASA and DPRA results, we also evaluated the use of EASA in two defined approaches (DAs) for skin sensitization hazard and compared it to that using DPRA (Table S2). Defined approaches, which are characterized by fixed data interpretation procedures applied to specific data sources, integrate data from multiple sources to make a decision on the classification of a substance [16]. We applied the “2 out of 3” DA and the KE 3/1 DA using either EASA or DPRA for one of the assays. The 2 out of 3 DA, first described in [56], predicts skin sensitization hazard by testing, in an undefined order, up to three non-animal methods that map to KEs 1, 2, and 3 of the AOP. The result of the DA is based on two concordant findings. We applied this DA using DPRA, KeratinoSens, and h-CLAT from the published literature (Table S2) and then compared the results to the DA using the EASA results in the place of DPRA. The KE 3/1 DA, first published in [57], is a simple decision tree that requires KE1 and KE3 data as inputs. If the results for an assay evaluating KE3 (h-CLAT in this study) indicates that the *TC* is positive, the *TC* is classified as positive. If the results for h-CLAT indicate that the substance is negative, then the substance is classified as positive or negative depending upon whether the result from an assay for KE1 (either EASA or DPRA in this study) is a binder or nonbinder, respectively. The performance of the DAs was calculated by determining accuracy, false negative and false positive rates with respect to LLNA results [58].

## 3. Results

### 3.1. Cause and Effect Analysis

**Figure 4:**
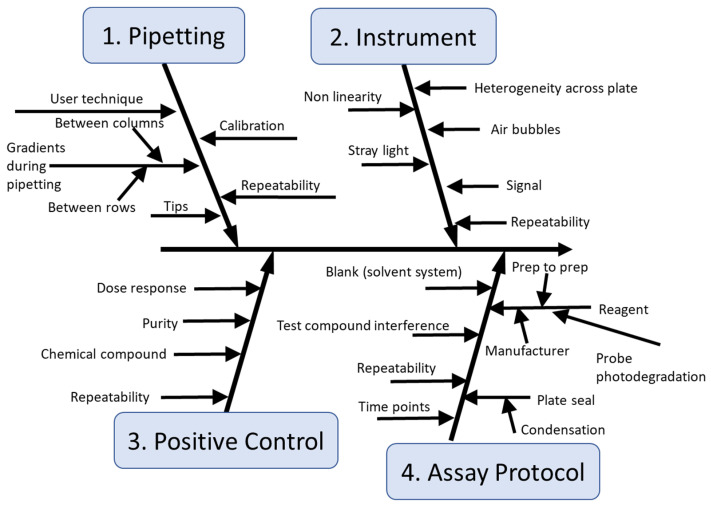
Cause-and-effect analysis of the EASA method. The four main branches indicate factors that are expected to have the greatest potential to cause variability in assay results.

A C&E analysis, an approach recently used with cellular and *Caenorhabditis elegans* toxicity assays [59,60,61,62,63,64,65], was performed to catalog what factors may be key sources of uncertainty for performing this assay using a 96-well plate format. The main branches of the C&E diagram indicate major potential sources of variability while the sub-branches describe the specific potential sources of uncertainty that collectively contribute to the main branches [60,63]. The C&E diagram (Figure 4) revealed four principal branches: pipetting; the instrument (in this case the plate reader); the PC; and the assay protocol. Some of the sources of variability in the pipetting branch are similar to those for the previously published C&E diagram for the 3-(4,5-dimethylthiazol-2-yl)-5-(3-carboxymethoxyphenyl)-2-(4-sulfophenyl)-2H-tetrazolium MTS cytotoxicity assay [60]. Since multichannel pipettes are used in each assay to add the probe molecule, it is important to quantify the pipetting variability within each pipetting step and between pipette steps for the volume added to the different wells. The instrument branch covers the potential for heterogeneity across the plate in the absorbance or fluorescence readings as a result of the plate reader performance and the potential for air bubbles to impact the absorbance or fluorescence readings. The third branch relates to the *PC* to assess the assay performance. Lastly, the assay protocol branch identifies many potential key sources of variability such as the potential for photodegradation of the probe molecule, issues with the plate seal such as condensation or degradation of the plate seal, and *TC* interference. Information from this diagram was used to develop a plate layout that incorporated in-process control measurements into the assay to provide information about the assay performance each time it is performed. In addition, other potential sources of variability identified by the C&E analysis were systematically evaluated in a series of experiments to assess the robustness of the assay.

### 3.2. Plate Layout and In-Process Control Measurements

From the C&E diagram, we designed a plate layout with several in-process control measurements (Figure 3). The first two in-process control measurements (Table S3), namely the within and between pipetting variability measurements, evaluate the reproducibility of the pipette within a single step and among steps. The third in-process control measurement relates to the signal pertaining to the SS (no probe molecule added). These wells should yield consistent, low absorbance or fluorescence values and are used for background subtraction for the *NC* and *PC* wells. The fourth in-process control measurement tests for *TC* interference, a measurement of the absorbance or fluorescence of the *TC* in the absence of the probe molecule. During preliminary testing, it was uncovered that many of the TCs at a concentration in the well of two mmol/L had a detectable absorbance or fluorescence signal at the same wavelengths as tested for the probe molecules or decreased the fluorescence signal for PDA. Therefore, these wells were used to quantify this source of bias and used to adjust for this when calculating the depletion for the TCs (see Equations (2) and (3)). The fifth in-process control measurement is for the dose-response value for the *PC* [66]. This curve yields information about the sensitivity of the assay to the same compound each time the assay is performed, similarly to a calibration curve for elemental analysis. In contrast to the approach frequently used in the toxicology field where only a single very high dose is tested for the *PC* [67,68], testing a full dose-response curve provides information about the assay performance by covering a range of responses that span the potential responses for TCs in the assay. This information can be helpful for comparing results among laboratories that are performing the test using different instruments, operators, etc. The last in-process control measurement in the assay protocol was designed to test if there were bubbles in the test wells that could impact the absorbance or fluorescence measurement in that well by measuring all wells at 680 nm (Feature # 6 in Table S3) [69], a wavelength outside of the absorbance spectrum of the probe molecules, shortly after the plate was added to the plate reader (i.e., within 5 min). When loading a plate using a pipette, it is unavoidable that air bubbles may unintentionally be created in some wells. Sometimes these can be manually eliminated, but other times, this is challenging, or the bubble is not visually evident during a cursory inspection. Nevertheless, it is possible that these bubbles could impact the plate reader measurements causing a bias in the determination of the percentage depletion. To evaluate the impact of bubbles on the assay results, plates were prepared with the SS in each of the wells, bubbles were induced by vigorously pipetting up and down, and the plates were evaluated at the absorbance and fluorescence wavelengths for the NBT and PDA assays.

### 3.3. Robustness Testing and Protocol Design

One-off control experiments were performed to evaluate the robustness of assay features that could affect the test results. A summary of the results for the robustness testing is provided in Table S4. Overall, the use of a 50:50 mixture of acetonitrile and phosphate buffer caused some complications that would not be observed in typical 96-well plate experiments that use an aqueous media, potentially with only a small concentration (1% or less) of organic solvent. This SS required the use of a more resistant type of plastic in the plate and plate seal as the SS was shown to degrade polystyrene plates and one of the microplate sealing tapes. In addition, it was necessary to keep a plate seal on the plate for the duration of the assay while the plate was in the plate reader to minimize the release of organic solvent into the plate reader, which could in turn cause degradation of the instrument and changes in the test chemical concentration.

One unexpected result from the robustness testing was the observation of NBT degradation during the course of plate loading when the assay was conducted under normal laboratory lighting conditions (see Figure S1). When a different light source was used for room lighting, in this case a red bulb, while the laboratory lights were turned off, photodegradation of the NBT reagent was minimized (Figure S1B). The approximately 10% higher absorbance readings for the wells in part B (red light used) compared to row 8 in part A (laboratory light used) suggests that photodegradation occurred even for the last row to which the NBT was added. To assess if there was a systematic trend among the pipetting steps for the NBT assay when red light was used and for the PDA fluorescence and absorbance assays, the values were compared for the *NC* wells for each column (Figure S2). A linear regression analysis of the PDA absorbance and fluorescence data did not show a trend that differed from zero, indicating the lack of a systematic trend. A statistically significant trend (*p* < 0.05) was observed for the NBT data even when the red light was used during loading the plate; however, the difference between the first and last column was less than 1% on average.

Another key topic for the robustness testing was the determination of an optimal assay duration. In the absence of degradation of the probe molecule or TC, longer durations may be superior as they could better enable the detection of weak sensitizers. However, in this study, we observed that the probe molecule concentration in the absence of TCs typically decreased with increasing reaction time (Figure S3), a finding most clearly observable for the PDA fluorescence assay results (Figure S3E) and for the NBT and PDA absorbance assays up to 50 min. In addition, we observed visually that there was condensation on the plate seal during prolonged reaction times (>1 h), likely as a result of evaporation and condensation of the acetonitrile; performing the assay at a higher temperature to accelerate the reaction would likely not be feasible as a result of condensation. This was also evident in increased mean and standard deviation values for the *NC* and SS wells after 50 min (Figure S3); this effect is believed to have counteracted the trend of decreasing NBT and PDA values with time observed during the first 50 min. However, the increase in the mean and standard deviation values was not observed for the PDA fluorescence results, suggesting that the condensation on the plate seal only impacted the absorbance results. When measuring the change in percentage depletion of the probe molecules across the *PC* dose-response curves for the PDA assays with time, there was not a clear increase in the percentage depletion caused by the *PC* at any concentration after 50 min (Figure S4). For the NBT assay, there were increases in the percentage depletion for the lowest concentrations of the *PC* after 50 min (Figure S4). It should be noted that these evaluations were only performed with a single *PC* and the optimal assay duration may differ for different mechanistic domains (e.g., Michael-type additions compared to SN2 reactions). During preliminary testing, it was determined that an increase in the uncertainty of the *NC* wells began to raise the statistical threshold to determine if a compound was a sensitizer as a result of condensation on the plate seal. Thus, there were tradeoffs when running the assay for longer durations: there would be a potential for a longer duration to yield a higher amount of depletion, but this may be offset by the decreased sensitivity of the assay as a result of the increasing uncertainty in assay results caused by an increased variability in the results among the wells. To minimize the impact of condensation on the plate seal while still having a nearly complete response for the PCs, an assay duration of 50 min was selected.

Absorbance data at 680 nm for the “bubble” test were collected from the *NC* or SS wells for 18 plates and analyzed (Table S5). In addition, a plate was prepared with just the SS in each of the wells and bubbles were intentionally added to the wells through vigorously pipetting up and down. Interestingly, some of the wells that had visually observable bubbles still yielded absorbance values at 680 nm that were near the background mode value (0.066), but most of the wells with bubbles did have an impact on the absorption readings (Figure S5). A tiny, hardly observable bubble located in the bottom center of a well, in addition to being impractical to remove, could have a substantial impact on the absorbance reading. In addition, the absorbance of the wells at 680 nm was compared to their absorbance or fluorescence values for the NBT and PDA assays (Figure S5). For the NBT and PDA absorbance assays, a linear slope was observed with a regression line that overlapped with one (R^2^ > 0.92), indicating a 1:1 correlation between the absorbance at these two wavelengths. Based on this information and the frequency distribution of absorbance values at 680 nm, an acceptance threshold was set at 0.081. Only 1% of the wells had values above this value. A 680 nm absorbance value just below the threshold (e.g., 0.079) would only lead to a 1–2% bias in the percentage depletion for that individual well for the NBT assay, thus setting a limit on the impact of outlier wells as a result of these bubbles on percentage depletion results. Given that these wells were infrequent (≈1% of wells), the actual bias on assay results will likely be substantially less since other wells for a particular *TC* are unlikely to be similarly biased as a result of inadvertent bubbles. For the PDA fluorescence assay, the results differed with a much weaker linear correlation between the fluorescence results (excitation at 324 nm and emission at 398 nm) and the bubble test results at 680 nm (Figure S5C). Setting the bubble test process control measurement at 5300 limited the maximum amount of bias from bubbles to less than 1%.

Another key source of potential bias in this assay was the potential for the absorbance or fluorescence signal from TCs to cause an interference by impacting measurements of the depletion of the probe molecules if the TCs had a similar absorbance or fluorescence signal or decreased the fluorescence signal in the *TC_blank_* wells. To address this potential source of bias, an in-process control measurement was added to the test plate for *TC* interference, and this was evaluated for all TCs. Overall, most of the TCs (79%, 71/90) had an interference effect with at least one of the three assays.

Five compounds were evaluated to assess the impact of using different solvents in the assay, namely the use of PB instead of acetonitrile to dissolve the TCs. This is necessary to determine the applicability domain for this assay since some TCs may not be soluble in semi-polar solvents such as acetonitrile. In addition, some agencies require performing extractions of products using different types of solvents (e.g., polar, semi-polar, and nonpolar) and then assessing the potential toxicity of the extracts. The use of phosphate buffer resolved issues of solubility for some of these compounds (e.g., squaric acid) and decreased the amount of *TC* interference observed for 4-phenylenediamine. For 4-phenylenediamine, the amount of interference observed for the PDA fluorescence readings when the substance was dissolved in acetonitrile caused the signal to be outside of the dynamic range of the instrument, while interference was not observed when PB was used.

### 3.4. Control Charting Results

Control charting is shown for the NBT, PDA absorbance, and PDA fluorescence assays in Figures S6, S7 and S8, respectively. Thresholds were set at three times the standard deviation of the mean values for all of the parameters: SS, NC, coefficient of variation for the NC, and IC_50_ values of the PC. For a normal distribution, values that are greater than three times the standard deviation from the mean would indicate values that are outside of approximately a 99% confidence interval. If a value for an in-process control measurement exceeded this threshold, the whole plate was determined to be an outlier and the data excluded (e.g., the PDA fluorescence plate on 4 May 2019 had a coefficient of variation value for the *NC* that was above the threshold; Figure S8C). A full list of specifications for this assay is provided in Table S6.

### 3.5. Test Substance Results

**Figure 5:**
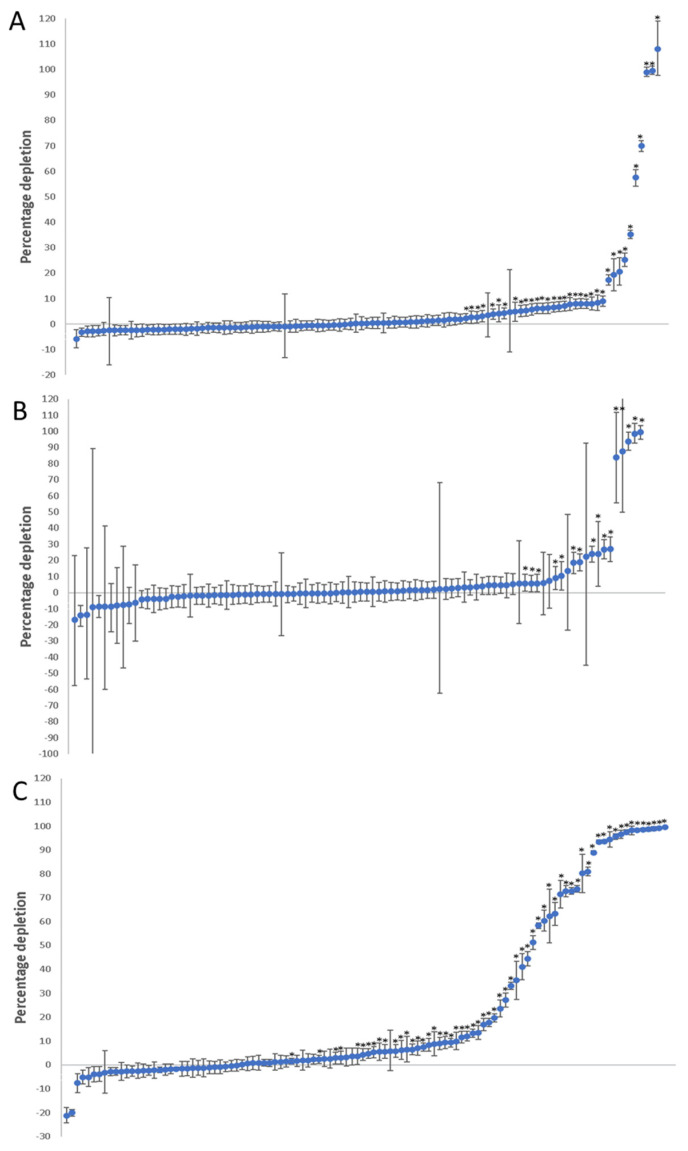
Values obtained from NBT (**A**), PDA absorbance (**B**), and PDA fluorescence (**C**) percentage depletion for test compounds. Data points and error bars indicate the mean and 95% confidence intervals determined using Bayesian modeling. Each data point represents the data for a *TC* in a particular run. Asterisks (indicated by *) indicate statistically significant results where the 95% confidence interval does not overlap with zero. Data is arranged from lowest to highest percentage depletion. Compounds with negative percentage depletion values less than −20% or percentage depletion values greater than 110% are excluded.

To evaluate the performance of this assay with a broad range of compounds and mixtures, a total of 92 TCs were analyzed (see Table 1 for a comparison of EASA with DPRA and in vivo results and Table S1 for a list of manufacturers and OASIS or OECD protein binding alerts from the OECD QSAR Toolbox). Results for these compounds were statistically evaluated using Bayesian and frequentist approaches (see Tables S7 through S12 for data on the individual TCs with each assay) and plotted in Figure 5. The PDA absorbance assay (Figure 5B) had the fewest compounds that showed a statistically significant amount of probe depletion (indicated by 95% confidence intervals that did not extend below zero based on a two-sided statistical test and α = 0.05), and substantially fewer than the NBT (Figure 5A) and PDA fluorescence assays (Figure 5C).

The results from EASA yielded agreement with LLNA and GPMT data of 73% using the frequentist modeling and α = 0.005 (Table 2). These results were similar to those for DPRA, which had an agreement of 77% (DPRA results for each *TC* are shown in Table 1). The level of agreement was similar for a range of α values (0.05, 0.01, 0.005, and 0.001) with the Bayesian modeling and the frequentist modeling (α = 0.005) (Table 2). However, there was a clear change in the number of false positive and false negative results with the false positive results decreasing and false negative results increasing with decreasing α. Interestingly, the percentage agreement with the LLNA data was in close agreement regardless of whether a Bayesian or frequentist statistical approach was used. Similar results were also obtained when using a threshold of five times the standard deviation (Table 2). When directly comparing the DPRA and EASA data, there was 73% agreement for the 44 TCs with DPRA data when using the frequentist modeling and an α value of 0.005.

The accuracy of the two out of three DAs were the same, 79%, regardless of whether EASA or DPRA was used as the KE1 assay (Table 3). The two out of three with EASA had balanced false negative and false positive rates, at 21%; however, the DA with DPRA had a higher false negative rate (28%) and lower false positive rate (8%). The accuracies of the KE 3/1 DAs were higher than those for the two out of three DAs. The accuracy for the KE 3/1 with EASA was 83%, while that for DPRA was 88%. The KE 3/1 with EASA had a higher false positive rate (46% vs. 8%) and a lower false negative rate (3% vs. 14%) than that with DPRA.

Results for the repeated analysis of the seven compounds with values that were close to the threshold of having statistically significant probe depletion for at least one of the three assays are provided in Tables S13 through S15. Overall, there was good agreement among the repeated results for all chemicals, except for pentaerythritol triacrylate with the NBT absorbance and PDA fluorescence assays, as shown by the repeated analyses of the same chemicals having 95% overlapping confidence intervals.

Performance against LLNA, calculated using Cooper statistics [58], are shown for the 44 chemicals with both EASA and DPRA results. The EASA results came from the statistical approach using the *t*-test and α = 0.005. The two out of three DA had two inconclusive results (D-glucose and isophorone diisocyanate) with DPRA and one inconclusive result (isophorone diisocyanate) with EASA. Isophorone diisocyanate lacked KeratinoSens and h-CLAT data. D-glucose lacked h-CLAT data and the KeratinoSens data were concordant with EASA, but not with DPRA. The KE 3/1 DA with EASA or DPRA had inconclusive results for D-glucose and isophorone diisocyanate due to the lack of h-CLAT data.

The Z-factors were similar for the NBT (0.89) and PDA fluorescence assays (0.92), and both were substantially larger than that for the PDA absorbance assay (0.62), indicating the superior sensitivity of the NBT absorbance and PDA fluorescence assays. In addition, the PDA absorbance data did not provide any additional information to determine whether a *TC* was identified as a sensitizer or non-sensitizer (Tables S7 through S9). Thus, the same overall results would have been obtained if the PDA absorbance assay had not been performed.

## 4. Discussion

Introducing robustness into an assay design requires an assessment of sources of variability and strategies to monitor and minimize the variability. We adopted a measurement science approach to both catalog potential sources of variability and design appropriate control experiments to understand their effect on test results and to minimize potential biases and artifactual results. For example, the light sensitivity of the NBT probe has the potential to lead to biased results, but this can be minimized by using the protocol developed and monitoring if there is a trend of decreasing values in the *NC* wells among the pipetting steps. Although the control measurements (Tables S3 and S4) are specifically designed for this assay, it is likely that they could be adopted to serve as control measurements in similar assays.

It is important to highlight that the 14 robustness testing measurements (Table S4) and the eight in-process control measurements systematically characterized all sources of uncertainty listed in the subbranches of the C&E diagram (Figure 4). Some of these sources of uncertainty were analyzed during robustness testing to optimize the protocol, while other sources of variability were tested through the in-process control measurements each time the assay was performed. This highlights one of the main benefits of developing a C&E diagram: it can guide the assay development process to ensure that all expected sources of variability are considered and possibly evaluated.

An important in-process control, especially for transferability of the assay, is the *PC* dose-response. The curve that results from the concentration dependence of the *PC* is based on the chemical reaction properties that also govern the chemical reactions between the probes and the TCs. Assuming the reaction rate constant follows the Arrhenius equation, the shape of the dose response curve will be dependent on temperature, activation energy for the reaction, and properties that govern the interaction between the components. If there are significant changes in the *PC* dose-response curve between experiments, which was not observed in this study conducted within a single laboratory, it suggests that properties of the assays that govern general chemical reactions may be different. Thus, the dose-response curve serves as an important assay system control for evaluating potential differences within and among laboratories (e.g., temperature of the room), in addition to revealing that probe reagents are reactive. This control also provides confidence in the consistency of the assay sensitivity during performance across time within a single laboratory. Whether this in-process control measurement should be tested periodically or every time the assay is performed depends upon tradeoffs between knowledge about the assay sensitivity each time it is performed and the economics of performing the assay with less frequent testing of this measurement. The greatest confidence in the assay results comes from testing this in-process control measurement each time the assay is performed. The *PC* dose-response data reveals information about the assay sensitivity, which can support the usage of this assay, or a revised assay format wherein dose-response curves for the TCs are evaluated, to assess the potency of TCs.

Another key in-process control measurement was the wells with the *TC* but without the probe molecule. These wells can be used to identify when the *TC* produces an absorption or fluorescence signal similar to that of the probe molecules (NBT or PDA). This potential bias was not described in the earlier cuvette-based studies for the EASA method [35]. Surprisingly, there were a substantial number (34%; 31/90) of TCs that caused a decrease in the fluorescence signal compared to the SS only wells. The agreement between TCs with interference and results from the LLNA animal assay (73% (40/55)) was similar to the results for all of the chemicals (77%; see Table 2). Thus, *TC* interference did not clearly cause a decrease in the capacity for this assay to yield results similar to those from LLNA. However, some TCs (4%, 4/90) did cause interferences so large in the PDA fluorescence assay that the measurements were no longer in the dynamic range of the instrument, thus necessitating a decrease in the *TC* concentration or the use of a different solvent to quench the interfering *TC* fluorescence. These chemicals are shown in Tables S7–S12 with the data labeled as not available. If the *TC* caused a significant amount of probe depletion at a lower concentration, this would yield a valid, positive result; however, if a compound is tested at a lower concentration (due to solubility or interference) and this resulted in no statistically significant depletion of the probe, that would be a valid result for that concentration but that would not exclude the potential for a positive result at a higher concentration. If the *TC* can be tested in another solvent at the higher concentration, the assay would still be valid at the higher concentration.

The *TC* and interference wells can also be used to help identify compounds that precipitate in the SS through the “bubble” measurements at 680 nm. One challenge in this test system is that the reaction products between the probe molecules and TCs are not removed prior to analysis, unlike for the liquid chromatography-based techniques such as DPRA. If the reaction products cause the formation of a precipitate, that may be detected by the “bubble” measurement. If the reaction products don’t cause a precipitate but do have an absorbance or fluorescence signal that overlaps with those of the probe molecules, that would increase the readings of the wells for the TC. We hypothesize that this type of interaction is what caused the anomalous results for 1-hydroxy-4-(p-toluidino) anthraquinone, which had a mean percentage depletion of −176%; the negative values indicated that there was an increase in signal in the *TC* wells relative to the control. When such results were observed in one of the assays, they were viewed as being inconclusive. However, it was not possible in this assay to detect if reaction products are produced with a signal similar to the amount of decrease in the probe molecule. This could lead to “false negatives” in this assay.

The average for the standard deviation of the *NC* percentage depletion was approximately 2-fold higher for the PDA absorbance (2.3%) compared to the NBT assays (1.3%) (Figures S6C and S7C). This result likely stems from the smaller difference between the mean SS and *NC* values for the PDA absorbance assay compared to the NBT assay as well as the lower mean absorbance value for the *NC* in the PDA absorbance assay. With smaller absorbance values, the instrument noise may have a larger impact. The PDA fluorescence assay had a similar percentage for the standard deviation of the *NC* (2.5%; Figure S8C) as the PDA absorbance assay.

The PDA fluorescence assay showed the largest number of compounds with significant probe depletion. This may stem from the lower concentration of PDA in the fluorescence assay (0.0064 mmol/L compared to 0.1 mmol/L for the PDA absorbance assay), increased sensitivity of the fluorescence measurement, and compounds that have interference with absorbance but not fluorescence. PDA fluorescence and absorbance results for the TCs are compared in Figure S9. Among the chemicals tested, there was only one *TC* (streptomycin sulfate) that showed a greater amount of probe depletion for the absorbance compared to the fluorescence assay; however, the assay yielded a “false positive” result for this TC. There are several chemicals that showed percentage depletion greater than 50% in the PDA fluorescence assay, which did not show significant probe depletion in the PDA absorbance assay. The difference in results between these assays is likely a result of the lower probe concentration for the PDA fluorescence assay. As this assay evaluates the relative change in a probe concentration, for the same total amount of probe molecule binding, it will be easier to detect a change for a lower probe concentration. In addition, the PDA absorbance assay consistently showed substantially larger 95% confidence intervals compared to the PDA fluorescence assay, a result again attributable to the lower PDA concentration for the PDA fluorescence assay. However, there are some compounds for which the 95% confidence intervals are smaller for the PDA absorbance assay as a result of stronger interference from the *TC* for fluorescence compared to that for absorbance. This leads to relatively more uncertainty in the final test result for the fluorescence values, given the larger values being compared between the *TC* and *TC* blank wells relative to the *NC* values.

The applicability domain of this assay is in some ways similar to DPRA in that prehaptens, prohaptens and metals would not be expected to be detected [34,35]. However, predictions for 11/12 pre-haptens tested in DPRA were reported to be binders in concordance with the positive LLNA results [70]. The following substances would also be outside the applicability domain of this assay: (1) substances that are not stable in the applicable solvents (solvent system or phosphate buffer) such as highly volatile substances or those that degrade in the solvents; (2) substances that are not soluble in the acetonitrile and phosphate buffer solvents; (3) substances that cause degradation of the plates, thereby interfering with the absorbance or fluorescence measures; and (4) substances that produce reaction products with the probe molecules that precipitate or cause an absorbance of fluorescence signal similar to those being used to quantify the probe molecules.

Among the statistical methods evaluated, the approach of using five times the standard deviation had a higher amount of false negative results compared to the Bayesian (α = 0.05) and frequentist approaches (Table 2). This finding indicates that five times the standard deviation is not a sufficient substitute for taking into account all relevant sources of uncertainty, such as the uncertainty for the *TC* or *TC_blank_*. Using a static call line (e.g., depletions > 10% are positive) would also not take into account all of the relevant sources of uncertainty.

Among the chemicals tested multiple times that had depletion values that were close to the threshold for determining if the *TC* was a potential sensitizer, only pentaerythritol triacrylate had repeated analyses where the 95% confidence intervals did not overlap with one another. This *TC* is highly viscous, which hindered reproducible pipetting; trimethylolpropane triacrylate was also highly viscous and had substantially different results in two NBT assays (Table S7). This analytical challenge combined with the low degree of probe depletion led to more variable results among the repeated experiments compared to those for the other chemicals. It is important to highlight that some chemicals, such as 5-amino-o-cresol, were close to the detection limit for the PDA fluorescence assay, yielding positive and negative results twice each in four different assays (using α = 0.005) (Table S15). This indicates that this assay was unable to provide a determination of the potential for this chemical to cause probe depletion with a high degree of confidence.

It is expected that chemicals with results close to the detection limit, sometimes referred to as the “borderline” region [71], would yield both positive and negative results some fraction of the times that the assay was tested. A similar finding for “borderline” substances was also observed for data from LLNA [72] and for other skin sensitization assays [71]. In this assay, “borderline” compounds close to the positive/negative determination were identified. A *TC* that is positive with less than 10% depletion was classified as a “potential” false positive (Type I error) in the data calculators, while a *TC* with greater than 3% depletion that was not statistically different from the *NC* (due to increased variability) was classified as a “potential” false negative (Type II error). These percentage depletion values do not correspond to a particular α value and were used to identify TCs for additional testing for the final determination. Labeling TCs with potential Type I and Type II errors will inform regulators that these TCs are likely to be at the borderline of the positive/negative determination at the tested concentration, allowing the regulator to make the proper assessment needed for their agency.

The results from these analyses and the robustness testing also enabled the development of test specifications for the in-process control measurements that will be assessed each time the assay is performed (Table S6). If the specification is not met, wells may need to be excluded (e.g., for the bubble test specification), or the plate may need to be repeated if the results indicate the plate is an outlier (e.g., *NC* mean value specification). There are also specifications such as for the observation of interference from the TC, which if not met, do not indicate that the assay needs to be repeated but instead that the results should be treated with caution and that additional testing may be recommended to confirm the results from the analyses conducted. A key topic for future evaluation during an ongoing interlaboratory comparison, during which each compound will be tested three times, is to evaluate when compounds should be analyzed additional times, potentially with a greater number of wells in each plate to decrease the variability, as a result of potential Type I and Type II errors. Based on the repeated analysis of the seven compounds, potential specifications include the retesting of chemicals with percentage depletion values less than 10%, which have significant results for probe depletion, or percentage depletion values greater than 3%, which do not have statistically significant results. Additional analysis is needed to identify a specification for compounds that do not have a statistically significant depletion result, but that have very large 95% confidence intervals extending above 10% (e.g., 4-hydroxychalcone in the NBT assay has 95% confidence intervals for the percentage depletion of −16.0% to 10.3%).

An analysis of results of almost 200 compounds for other skin sensitization assays yielded agreements with LLNA results of 76% (*n* = 194), 77% (*n* = 188), and 81% (*n* = 166) for DPRA, Keratinosens, and h-CLAT, respectively [37]. The concordance of EASA and LLNA results for the compounds tested in this study 77% (*n* = 64) was similar to those results. The EASA concordance results in this study were similar to those for DPRA, a commonly used standard method for analyzing the protein binding step of the skin sensitization AOP. It should be noted that EASA had a higher false positive rate (40%) than DPRA when compared directly to LLNA, although the overall concordance is similar. For some regulatory purposes, it may be preferable to have a higher false positive rate and a lower false negative rate since fewer skin sensitizers would be missed. The performance of EASA in two DAs for skin sensitization hazard yielded similar accuracy to the same DAs with DPRA, but the DAs with EASA tended to have higher false positive rates and lower false negative rates (Table 3).

Additional testing is needed with a broader range of compounds to more fully evaluate EASA performance. One option for this testing would be after adapting this assay to a robotic high-throughput screening system to facilitate the testing of a much greater number of compounds than could be readily achieved by manual testing. Even with manual testing using 96-well plates, the throughput of this assay is substantially higher than DPRA with the ability to test up to 14 TCs daily with the NBT and PDA fluorescence assays. Comparing results from EASA to assays other than DPRA [73,74] that also evaluate KE1, would also be valuable. A key topic for additional research will be to assess if this assay, or a modification of it, that tests a *TC* in a dose-response format similar to the kinetic DPRA assay [75,76], could predict *TC* potency (i.e., Globally Harmonized System of Classification and Labelling of Chemicals (GHS) sub-categories). Another next step is to test the transferability of this assay with interlaboratory testing to more vigorously evaluate the protocol. Evaluating this assay using a broader range of TCs including those with human skin sensitization data is another important future direction. The highly curated reference data used to evaluate the defined approaches in OECD Guideline 497 would be appropriate as human reference data are provided for 66 chemicals and LLNA reference data are provided for 168 chemicals [32,39].

## Figures and Tables

**Table 1:** Description of test chemicals including results from other studies and overall EASA results.

Chemical Name	CAS	DPRA Result	Reference ^a^	In Vivo Result	Reference ^b^	In Vivo Assay	*LLNA EC_3_* ^c^	*EASA* ^d^
1,2-Propanediol	57-55-6	Nonbinder	[37]	Nonsensitizer	[37]	LLNA		Binder
12-Bromo-1-dodecanol	3344-77-2		[37]	Sensitizer	[37]	LLNA	6.9%	Nonbinder
1-Butanol	71-36-3	Nonbinder	[37]	Nonsensitizer	[37]	LLNA		Nonbinder
1-Butyl-1-methylpyrrolidinium chloride	479500-35-1							Nonbinder
1-Butyl-3-methylimidazolium chloride	79917-90-1							Nonbinder
1-Ethyl-3-methylimidazolium chloride	65039-09-0							Nonbinder
1-Hydroxy-4-(p-toluidino) anthraquinone	81-48-1							Binder
2,3-Butanedione	431-03-8	Binder	[37]	Sensitizer	[37,38]	LLNA	11%	Binder
2,4,5-Trichlorophenoxyacetic acid	93-76-5			Sensitizer	[39]	LLNA	9.87%	Nonbinder
2,4-Diaminotoluene	95-80-7			Sensitizer	[40]	LLNA	19%	Binder
2,4-Dichloronitrobenzene	611-06-3			Sensitizer	[41]	LLNA		Binder
2-Amino-6-chloro-4-nitrophenol	6358-09-4			Sensitizer	[38,42]	LLNA	2.2%	Binder
2-Mercaptobenzothiazole	149-30-4	Binder	[37]	Sensitizer	[37]	LLNA	1.7%	Binder
2-Methoxy-4-nitroaniline	97-52-9			Nonsensitizer	[39]	GPMT		Binder
2-Methyl-4H,3, 1-benzoxazin-4-one (Product 2040)	525-76-8			Sensitizer	[37]	LLNA	0.7%	Inconclusive
3,4-Dihydrocoumarin	119-84-6	Binder	[37]	Sensitizer	[37]	LLNA	5.6%	Binder
3-Iodo-2-propynyl butylcarbamate	55406-53-6	Binder	[37]	Sensitizer	[37]	LLNA	0.9%	Binder
4-Chloro-o-phenylenediamine	95-83-0							Binder
4′-Hydroxychalcone	2657-25-2			Sensitizer	[42]	LLNA	0.002%	Binder
4-Methylcyclohexanemethanol	34885-03-5							Nonbinder
4-Phenylenediamine	106-50-3	Binder	[37]	Sensitizer	[37]	LLNA	0.16%	Binder
5-Amino-o-cresol	2835-95-2	Binder	[37]	Sensitizer	[43]	LLNA	3.4%	Binder
Ammonium thiosulfate	7783-18-8			Nonsensitizer	[39]	LLNA		Nonbinder
Aniline	62-53-3	Nonbinder	[37]	Sensitizer	[37]	LLNA	0.9%	Binder
Annatto	1393-63-1			Sensitizer	[44]	LLNA	5%	Binder
Atrazine	1912-24-9			Sensitizer	[39]	LLNA	31.3% to 41.4%	Nonbinder
Azithromycin	83905-01-5							Binder
Benzalkonium chloride	8001-54-5	Nonbinder	[37]	Nonsensitizer	[37]	LLNA		Nonbinder
Benzethonium chloride	121-54-0							Nonbinder
Benzyl benzoate	120-51-4	Nonbinder	[37]	Sensitizer	[37]	LLNA	17%	Nonbinder
Benzyl bromide	100-39-0	Binder	[37]	Sensitizer	[37,38]	LLNA	0.2%	Binder
Benzyl salicylate	118-58-1	Nonbinder	[37]	Sensitizer	[37]	LLNA	2.9%	Binder
Camphorquinone	10373-78-1			Sensitizer	[38]	LLNA	10%	Binder
Chlorpyrifos	2921-88-2			Sensitizer	[39]	LLNA	6.91%	Binder
Cinnamic aldehyde	104-55-2	Binder	[37]	Sensitizer	[37]	LLNA	3.1%	Binder
Cinnamyl Alcohol	104-54-1	Binder	[37]	Sensitizer	[37]	LLNA	21%	Binder
cis-Bixin	6983-79-5			Sensitizer	[44]	LLNA	0.1%	Binder
Citral	5392-40-5	Binder	[37]	Sensitizer	[37]	LLNA	4.6% to 13%	Binder
Clarithromycin	81103-11-9							Binder
D-glucose	50-99-7	Binder	[37]	Nonsensitizer	[37]	LLNA		Nonbinder
Dicyclohexylcarbodiimide	538-75-0							Binder
Diethyl maleate	141-05-9	Binder	[37]	Sensitizer	[37]	LLNA	2.1%	Nonbinder
Dinitrochlorobenzene	97-00-7	Binder	[37]	Sensitizer	[37]	LLNA	0.04%	Binder
Ethyl vanillin	121-32-4	Nonbinder	[37]	Nonsensitizer	[37]	LLNA		Binder
Ethylene thiourea	96-45-7							Nonbinder
Fluconazole	86386-73-4							Binder
Formaldehyde	50-00-0	Binder	[37]	Sensitizer	[38]	LLNA	0.61%	Binder
Furil	492-94-4	Binder	[37]	Nonsensitizer	[37]	LLNA		Binder
Glutaraldehyde	111-30-8	Binder	[37]	Sensitizer	[37,38]	LLNA	0.1%	Binder
Glycerol	56-81-5	Nonbinder	[37]	Nonsensitizer	[37,38]	LLNA		Nonbinder
Glyoxal	107-22-2	Binder	[37]	Sensitizer	[37,38]	LLNA	1.4%	Binder
Heptachlor (solution)	76-44-8							Nonbinder
Iso-E Super	54464-57-2			Sensitizer	[45]	LLNA		Binder
Isophorone diisocyanate	4098-71-9	Binder	[37]	Sensitizer	[37,46]	LLNA	0.1%	Binder
Isopropanol	67-63-0	Nonbinder	[37]	Nonsensitizer	[37]	LLNA		Nonbinder
Methyl pyruvate	600-22-6	Nonbinder	[37]	Sensitizer	[37]	LLNA	2.4%	Binder
Methyl salicylate	119-36-8	Nonbinder	[37]	Sensitizer	[38,47]	LLNA		Binder
*N*,*N*-Diethyl-m-aminophenol	91-68-9			Sensitizer	[38]	LLNA		Binder
*N*,*N*-Dimethylformamide	68-12-2	Nonbinder	[37]	Nonsensitizer	[37]	LLNA		Nonbinder
o-Benzyl-p-chlorophenol	120-32-1							Binder
o-Cresol	95-48-7							Binder
p,p′-Biphenol	92-88-6							Inconclusive
Palladium di(4-oxapent-2-en-2-oate)	14024-61-4							Binder
Penicillin	61-33-6	Binder	[37]	Sensitizer	[37]	LLNA	30%	Nonbinder
Pentaerythritol triacrylate	3524-68-3							Binder
Perillaldehyde	2111-75-3	Binder	[37]	Sensitizer	[37]	LLNA	8.1%	Binder
Phenylacetaldehyde	122-78-1	Binder	[37]	Sensitizer	[37]	LLNA	3% to 4.7%	Binder
Potassium dicyanoaurate	13967-50-5							Nonbinder
Pyridine	110-86-1	Nonbinder	[37]	Sensitizer	[37,38]	LLNA	71.2%	Nonbinder
Pyrogallol	87-66-1			Sensitizer	[44]	LLNA	0.4% to 1.4%	Binder
R-Carvone	6485-40-1	Binder	[37]	Sensitizer	[37]	LLNA	12.9%	Binder
Resorcinol	108-46-3	Nonbinder	[37]	Sensitizer	[37]	LLNA	5.92%	Nonbinder
Saccharin	81-07-2	Nonbinder	[37]	Nonsensitizer	[37]	LLNA		Nonbinder
Sodium dodecyl sulfate	151-21-3	Inconclusive	[37]	Sensitizer	[37]	LLNA	14%	Nonbinder
Sodium metasilicate	6834-92-0			Nonsensitizer	[48]	LLNA	2% to 6%	Inconclusive
Sodium octyl sulfate	142-31-4							Nonbinder
Squaric acid	2892-51-5	Binder	[37]	Sensitizer	[37,38,49]	LLNA	4.3%	Binder
Streptomycin sulfate	3810-74-0	Nonbinder	[37]	Nonsensitizer	[37]	LLNA		Binder
Sulfanilamide	63-74-1	Nonbinder	[37]	Nonsensitizer	[37,38]	LLNA		Nonbinder
Tetraethylthiuramdisulfide	97-77-8			Sensitizer	[38]	LLNA	5.2%	Binder
Tetramethylthiuram disulfide	137-26-8	Binder	[37]	Sensitizer	[37]	LLNA	3.1%	Binder
Tetramethylthiurammonosulfide	97-74-5			Sensitizer	[50]	LLNA	5.4%	Binder
trans-2-Hexenal	6728-26-3	Binder	[37]	Sensitizer	[37]	LLNA	5.5%	Binder
trans-p-Hydroxycinnamic acid	501-98-4							Binder
Triethanolamine	102-71-6			Nonsensitizer	[39]	GPMT		Binder
Trimethylolpropane triacrylate	15625-89-5			Sensitizer	[39]	LLNA	0.01% to 0.13%	Inconclusive
Tri-n-octylphosphine oxide	78-50-2							Binder
Triphenyl phosphate	115-86-6			Nonsensitizer	[39]	GPMT		Nonbinder
Tween 80	9005-65-6	Nonbinder	[37]	Nonsensitizer	[37]	LLNA		Binder
Vanillin	121-33-5	Inconclusive	[37]	Nonsensitizer	[37]	LLNA		Binder
Zinc diethyldithiocarbamate	14324-55-1			Sensitizer	[50]	LLNA	0.2%	Binder
α-Hexylcinnamaldehyde	101-86-0	Nonbinder	[37]	Sensitizer	[37]	LLNA	12%	Binder

LLNA indicates local lymph node assay. DPRA indicates direct peptide reactivity assay. EASA indicates electrophilic allergen screening assay. GPMT indicates the guinea pig maximization test. ^a^ The references in this column are the citations for the DPRA data. ^b^ The references in this column are the citations for the LLNA data. ^c^ EC_3_ refers to the concentration that induces a stimulation index of three. However, this data was only available for some TCs. ^d^ The overall EASA results were determined using frequentist approach with a *t*-test and α = 0.005.

**Table 2:** Summary of concordance (agree, false positive, and false negative results) for EASA results and those from animal studies.

	Bayesian α = 0.05	Bayesian α = 0.01	Bayesian α = 0.005	Bayesian α = 0.001	Frequentist *t*-Test α = 0.005	5 × Standard Deviation	DPRA
*GPMT and LLNA data*							
Agree	70% (47/67)	71% (46/65)	69% (45/65)	65% (45/69)	73% (49/67)	70% (46/66)	77% (34/44)
False Positive	53% (9/17)	47% (7/15)	47% (7/15)	35% (6/17)	47% (8/17)	32% (6/19)	15% (2/13)
False Negative	22% (11/50)	24% (12/50)	26% (13/50)	36% (18/50)	20% (10/50)	30% (14/47)	26% (8/31)
*Only LLNA data*							
Agree	73% (47/64)	73% (46/63)	71% (45/63)	67% (44/66)	77% (49/64)	71% (44/62)	77% (34/44)
False Positive	47% (7/15)	43% (6/14)	43% (6/14)	33% (5/15)	40% (6/15)	31% (5/16)	15% (2/13)
False Negative	20% (10/49)	22% (11/49)	24% (12/49)	35% (17/49)	18% (9/49)	28% (13/47)	26% (8/31)

Three chemicals (2-Methoxy-4-nitroaniline, Triethanolamine, and Triphenyl phosphate) only had guinea pig maximization test (GPMT) data and were excluded from the local lymph node assay (LLNA) comparison. Comparisons were performed using Bayesian analysis, a *t*-test, or by comparing the mean percentage depletion value to five times the standard deviation of the negative control. The GPMT, LLNA, and DPRA data are provided in Table 1. The *t*-test α = 0.005 results shown here match the binder/nonbinder determinations in Table 1.

**Table 3:** Performance of defined approaches with EASA or DPRA with LLNA as the reference.

Performance Statistic	2 out of 3 with EASA	2 out of 3 with DPRA	KE 3/1 with EASA	KE 3/1 with DPRA
Accuracy	79% (34/43)	79% (33/42)	83% (35/42)	88% (37/42)
False Positive	21% (3/14)	8% (1/13)	46% (6/13)	8% (1/13)
False Negative	21% (6/29)	28% (8/29)	3% (1/29)	14% (4/29)

## Data Availability

Data from conducting the EASA method with the different TCs is provided in the Supplementary Materials.

## Supplementary Materials

The following supporting information can be downloaded at: https://www.mdpi.com/article/10.3390/toxics10050257/s1. Supplementary methods: 4-Nitrobenzenethiol (NBT assay procedure), Pyridoxylamine (PDA) Absorbance Assay Procedure, PDA Fluorescence Assay Procedure, Phosphate Buffer Procedure, Electrophilic Allergen Screen Assay (EASA) Safe Operating Procedure; Figure S1. Photosensitivity, Figure S2. Pipetting Robustness Test, Figure S3. Negative Controls, Figure S4. Positive Controls, Figure S5. Bubble Test, Figure S6. Control charting data for the NBT assay, Figure S7–Control charting data for the PDA absorbance assay, Figure S8. Control charting data for the PDA fluorescence assay, Figure S9. Comparison of values obtained from the PDA absorbance and PDA fluorescence percentage depletion for test compounds, Table S1. Chemicals and Alerts, Table S2. New and Historical Chemical Data, Table S3. Summary of in-process control measurements in plate design, Table S4. Summary of robustness testing results, Table S5. Bubble Test Data, Table S6. Specifications for the EASA assay, Table S7. Results from testing the test compounds with the 4-nitrobenzenethiol (NBT) absorbance assay analyzed using a Bayesian model, Table S8. Results from testing the test compounds with the pyridoxylamine (PDA) absorbance assay analyzed using a Bayesian model, Table S9. Results from testing the test compounds with the pyridoxylamine (PDA) fluorescence assay analyzed using a Bayesian model, Table S10. Results from testing the test compounds with the 4-nitrobenzenethiol (NBT) absorbance assay analyzed using a frequentist model, Table S11. Results from testing the test compounds with the pyridoxylamine (PDA) absorbance assay analyzed using a frequentist model, Table S12. Results from testing the test compounds with the pyridoxylamine (PDA) fluorescence assay analyzed using a frequentist model, Table S13. NBT Bayesian Analysis, Table S14. PDA Absorbance Bayesian, Table S15. PDA Fluorescence Bayesian Analysis; Supplementary Spreadsheets: NBT Data Calculator, PDA Fluor Data Calculator, Raw Data for NBT, Raw Data for PDA Absorbance, Raw Data for PDA Fluorescence, Example of Completed Worksheet.
