# Supplementary material for: Development of a 96-Well Electrophilic Allergen Screening Assay for Skin Sensitization Using a Measurement Science Approach
Source: Toxics. 2022 May 17;10(5):257. doi: 10.3390/toxics10050257 (PMC9147637; doi:10.3390/toxics10050257)
Supplement: Supplementary file 1 [file toxics-10-00257-s001.zip › toxics-1685862-supplemental material.pdf]

toxics MDPI

Supplementary Materials: Development of a 96-Well Electrophilic Allergen Screening Assay for Skin Sensitization Using a Measurement Science Approach

Elijah J. Petersen, Richard Uhl, Blaza Toman, John T. Elliott, Judy Strickand, James Truax and John Gordon

Supplemental methods

NIST disclaimer: Certain instruments or materials are identified in this paper in order to adequately specify experimental details. In no case does it imply endorsement by NIST or imply that it is necessarily the best product for the experimental procedure.

Standard Operation Procedures (SOPs)

4-Nitrobenzenethiol (NBT) Assay Procedure

1) Turn on the plate reader, pull up (or set up if first run) the NBT protocol with a wavelength of 412 nm, and allow the instrument to warm up/stabilize according to manufacturer's guidelines.
   a. It is recommended to set up the plate reader protocol using a kinetic run. This will keep the plate in the reader for the entire run. Consecutive timed readings may open the reader and bring the plate into the light.
   b. Set up plate reader protocol to take an initial reading "at time zero" (which is actually 5 min after adding the probe molecule) and then to read every 15 min to end at 50 min after addition of the probe molecule.
2) Make Solvent System (SS) by mixing equal volumes of PB (0.1 mol/L, pH 7.4 ±0.2) with acetonitrile (ACN) to make a 50:50 solution. Volumes can be adjusted for the number of tests to be run. Stock can be stored at room temperature for 30 days.
3) Make a 1 mmol/L NBT Stock solution (MW 155.17 g/mol) in SS. Sonicate for approximately 1 min to 2 min to aid dissolution. This solution is stable when stored up to 7 days at -20° ± 2°C and protected from light (amber vials and/or wrapping in aluminum foil is recommended). Aliquot into smaller volumes for daily use to avoid repeated freezing and thawing.
   a. Note: oxidized NBT powder has occasionally been shipped from the chemical supplier.
4) Dilute NBT stock solution (Step 3) 1:8 with SS (Step 2) for the NBT Working solution (0.125 mmol/L).
   a. Example: 3 mL of 1 mmol/L NBT Stock + 21 mL SS. Enough Working solution should be prepared for the samples to be run on a plate.
   b. Protect the solution from light (amber vials and/or wrapping in aluminum foil is recommended); if possible, use only red light to prepare the NBT Working solution.
5) Make solutions of the TCs in ACN.
6) Make a 3 mmol/L solution of the positive control benzyl bromide (BB) in ACN.
7) Load the 96-well plate according to the plate layout below (Figure 2). The NBT Working solution (0.125 mmol/L) must be added last and the time for incubation started when the first row or column of NBT is added. <u>Note</u>: The total volume for each well is 200 µL.
   a. Negative control (NC)/positive control (PC) Blanks – 40 µL ACN (Column 1)
   b. NC – 40 µL ACN (Column 2 and Row A)

c. PC –Seven triplicate 1:2 serial dilutions of BB starting with 40 µL 3 mmol/L BB stock solution in Columns 3 – 5, Row B, with the serial dilution ending in Columns 3 – 5, Row H. The final volume in each well must be 40 µL.
d. TC - 40 µL of the TC to columns 6 – 12 horizontally starting in Row B

8) Add 160 µL of SS to columns 1, 10, 11, and 12.
9) **Turn on Red light, and turn off all lab lights**. Immediately add 160 µL of the NBT Working solution (0.125 mmol/L) to columns 2 – 9 vertically using a multi-channel pipette adding vertically from left to right. Do not add NBT Working solution to columns 1, 10, 11, and 12. Start the timer when the first column of NBT working solution is added.
   a. Note: Try to locate an area that has no ambient light that could affect the NBT.
   b. Examine the plate for bubbles.
      i. Tip: Bubbles can be removed using a 10 µL pipette tip.
10) Place VIEWseal plate seal on plate.
11) Record absorbance at approximately 5 min ± 30 s, 20 min ± 2 min, 35 min ± 2 min, and 50 min ± 2 min, and calculate relative absorbance at each time point. The start time should begin when the NBT working solution is first added.

Pyridoxylamine (PDA) Absorbance Assay Procedure

1) Turn on the plate reader, and pull up the PDA Absorbance protocol (or set the protocol up if it is the first run). Be sure the wavelength is set to 324 nm. Allow the instrument to warm up/stabilize according to manufacturer's guidelines.
   a. Set up plate reader protocol to take an initial reading "at time zero" (which is actually 5 min after adding the probe molecule) and then to read every 15 min, to end at 50 min after addition of the probe molecule.
2) Make Solvent System (SS) by mixing equal volumes of PB (0.1 mol/L, pH 7.4 ±0.2) with ACN to make a 50:50 solution. Volumes can be adjusted for the number of tests to be run. Stock can be stored at room temperature for 30 days.
3) Make a 1 mM Stock solution of PDA (MW 241.11 g/mol) in SS (stable for 7 d at room temperature).
4) Dilute PDA stock solution (Step 3) 1:8 with SS (Step 2) for the 0.125 mmol/L PDA Absorbance Working solution.
   a. Example: 3 mL PDA Stock Solution + 21 mL SS. Enough stock solution should be prepared for each plate produced.
5) Make a solution of the TCs, according to the chemical-specific instructions provided, in ACN.
6) Make a 1 mmol/L solution of the PC (Glutaraldehyde) in ACN.
7) Load the 96-well plate according to the plate layout below (Figure 2). The PDA Absorbance Working (0.125 mmol/L) solution should be added last and the time for incubation started when the first row or column of PDA working solution is added. Note: The total volume for each well is 200 µL.
   a. NC/PC Blanks – 40 µL ACN (Column 1)
   b. NC – 40 µL ACN (Column 2 and row A)
   c. PC – Seven triplicate 1:2 serial dilutions starting with 40 µL 1 mmol/L Glutaraldehyde in Column 3, Row B and ending in Column 5, Row H. The final volume in each well must be 40 µL.
   d. TC – 40 µL of the TC (in columns 6 – 12 horizontally starting in Row B)
8) Add 160 µL of SS to columns 1, 10, 11, and 12.
9) Immediately add 160 mL of PDA Absorbance Working solution (0.125 mmol/L) to columns 2 – 9. Add the PDA working solution vertically from left to right using a multi-channel pipette. Do not add PDA working solution to columns 1, 10, 11, and 12. Start the timer when the first column of PDA working solution is added.
   a. Examine the plate for bubbles.
      i. Tip: Bubbles can be removed using a 10 µL pipette tip
10) Place VIEWseal™ plate seal on plate.

11) Record absorbance at approximately 5 min ± 30 sec, 20 min ± 2 min, 35 min ± 2 min, and 50 min ± 2 min. Start timing after the first addition of PDA working solution.

PDA Fluorescence Assay Procedure

1) Turn on the plate reader and pull up PDA Fluorescence protocol (or set up the protocol if first time). Make sure to set the excitation wavelength to 324 nm and the emission wavelength to 398 nm. Allow the instrument to warm up/stabilize according to manufacturer's guidelines.
   a. Set up plate reader protocol to take an initial reading "at time zero" (which is actually 5 min after adding the probe molecule) and then to read every 15 min, to end at 50 min after addition of the probe molecule.
2) Make Solvent System (SS) by mixing equal volumes of PB (0.1 mol/L, pH 7.4 ±0.2) with ACN to make a 50:50 solution. Volumes can be adjusted for the number of tests to be run. Stock can be stored at room temperature for 30 d.
3) Make 1 mmol/L PDA stock solution (MW 241.11 g/mol) in SS (stable for 7 d at room temperature).
4) Make a PDA Fluorescence Working solution (0.008 mmol/L) by adding 200 µL of PDA stock (Step 3) to 25 mL SS (Step 2).
5) Make a 0.1 mmol/L solution of the PC (Glutaraldehyde) in ACN.
6) Prepare the TC, according to the chemical-specific instructions provided, in ACN.
7) Load the 96-well plate according to the plate layout below (Figure 2). The PDA Fluorescence Working solution must be added last and the time for incubation started when the first row or column of PDA is added. Note: The total volume for each well is 200 µL.
   a. NC/PC Blanks – 40 µL ACN (Column 1)
   b. NC - 40 µL ACN (Column 2 and Row A)
   c. PC – Seven triplicate 1:2 serial dilutions of the PC, starting with 40 µL 0.1 mmol/L Glutaraldehyde stock solution in Columns 3 – 5, Row B, with the serial dilutions ending in Columns 3 – 5, Row H. The final volume in each well must be 40 µL.
   d. TC - 40 µL of the TC to columns 6 – 12 horizontally starting in Row B
8) Add 160 µL of SS to columns 1, 10, 11, and 12.
9) Immediately add 160 µL of PDA Fluorescence Working solution to columns 2 – 9. Do not add PDA working solution to columns 1, 10, 11, and 12. Start the timer when the first column of PDA working solution is added.
   a. Examine the plate for bubbles.
      i. Tip: Bubbles can be removed using a 10 µL pipette tip.
10) Place VIEWseal plate seal on plate.
11) Read the fluorescence (Fluor) at approximately 5 min ± 30 s, 20 min ± 2 min, 35 min ± 2 min, and 50 min ± 2 min. Start the timer when the PDA working solution is first added.

Phosphate Buffer Procedure

All steps in the protocols are performed similarly, except that phosphate buffer is used in place of acetonitrile in all steps. In addition, the maximum concentration of glutaraldehyde for the PDA fluorescence assay was decreased by a factor of two in step 5 to 0.0.05 mmol/L, and the PDA working solution concentration was decreased to 0.0024 mmol/L.

Electrophilic Allergen Screen Assay (EASA) Safe Operating Procedure

Note: This procedure serves as an example for other laboratories. The safety procedures outlined here are specific to NIST. Each laboratory should consider additional safety measures, as applicable to their specific operational needs.

1. Purpose

a. To describe the hazards and required control measures for the EASA protocol.

2. Background and Hazards

a. The EASA protocol is a set of procedures that measures loss of absorbance and/or fluorescence of 4-Nitrothiophenol (NBT) and Pyridoxamine dihydrochloride (PDA) with covalent binding by electrophilic haptens (chemical allergens) at 25 °C with respect to hapten concentration and time.
b. The primary hazards associated with the EASA protocol are the specific health and physical hazards of the chemicals involved in the protocol. The following is a summary of the hazard classes presented by the chemicals used in the EASA protocol:
   (1) Acute aquatic toxicity (Category 1-3), H400-402
   (2) Acute toxicity, Dermal (Category 2-4), H310-312
   (3) Acute toxicity, Inhalation (Category 3-4), H331-332
   (4) Acute toxicity, Oral (Category 2-4), H300-302
   (5) Carcinogenicity (Category 1A-2), H350-351
   (6) Chronic aquatic toxicity (Category 1-3), H410-412
   (7) Eye irritation (Category 2A), H319
   (8) Flammable liquids (Category 2-4), H225-227
   (9) Germ cell mutagenicity (Category 2), H341
   (10) Respiratory sensitisation (Category 1), H334
   (11) Serious eye damage (Category 1), H318
   (12) Skin corrosion (Category 1B), H314
   (13) Skin irritation (Category 2), H315
   (14) Skin sensitisation (Category 1), H317
   (15) Specific target organ toxicity - repeated exposure (Category 2), H373
   (16) Specific target organ toxicity - single exposure (Category 1), H370
   (17) Specific target organ toxicity - single exposure (Category 3), Central nervous system, H336
   (18) Specific target organ toxicity - single exposure (Category 3), Respiratory system, H335

3. Materials

a. Equipment
   (1) Bath sonicator
   (2) 20 mL borosilicate containers with Teflon-lined caps
   (3) Weighing paper
   (4) Static gun
   (5) Microbalance
   (6) Balance
   (7) Plate reader
   (8) 96 well plates
b. Chemical Identity and Hazards
   (1) Test Chemicals (TCs)
      (a) As a default, chemicals should be treated as if they are extremely hazardous or potentially carcinogenic.
   (2) Chemicals in assay
      (a) 4-Nitrobenzenethiol (NBT) (CAS # 1849-36-1): causes skin and eye irritation, may cause respiratory irritation
      (b) Pyridoxylamine dihydrochloride (CAS # 524-36-7): no hazards listed
   (3) Positive Controls (PCs)
      (a) For NBT assay, benzyl bromide (BB) (CAS # 100-39-0): causes skin and eye irritation and may cause respiratory irritation.
      (b) For PDA assay, glutaraldehyde (CAS # 111-30-8): harmful if swallowed or inhaled, may cause severe skin or eye damage, very toxic to aquatic life.
   (4) Solvents

- (a) Water (no hazards)
- (b) Phosphate buffer (no hazards)
- (c) Acetonitrile (CAS #: 75-05-8): highly flammable, harmful if swallowed, causes serious eye irritation.

4. Hazard Controls

a. Minimization
   - (1) Minimize the chemical quantities in use and storage to reduce hazards.

b. Engineering Controls
   - (1) Use a properly functioning chemical fume hood when working with a particularly hazardous substance (PHS) that presents an inhalation hazard (*e.g.*, gas, vapor, dust, or mist) or that generates flammable or toxic gases upon contact with other chemicals or materials in the work area. PHSs are chemicals classified with any of the following hazard classes: acutely toxic, germ cell mutagenicity, respiratory or skin sensitization, and specific target organ toxicity-single exposure) Work with the fume hood sash as far down as feasible, and keep chemical containers at least 6 inches behind the fume hood sash plane.

c. Administrative Controls/Work Practices
   - (1) Identify all PHSs associated with an activity (reactants, reaction media, products, and byproducts).
   - (2) Design your activity to minimize the amount of chemical you need to use and to avoid exposure from splashes, drips, sprays, and mists. Minimize PHS quantities outside of storage.
   - (3) Ensure the following required controls are documented in the associated hazard review, this document, and implemented for the activity:
     - (a) Establishment of a designated area;
     - (b) Use of containment devices, such as fume hoods or a Nanoenclosure;
     - (c) Procedures for safe removal of contaminated waste; and,
     - (d) Decontamination procedures.
   - (4) Ensure employees and associates have been informed of the following:
     - (a) The labeling system employed at the workplace; and
     - (b) How to obtain access to the Hazardous Chemical Inventory List and SDSs for the hazardous chemicals in the work area.
   - (5) Perform work only after the work and worker have been authorized in accordance with all applicable laboratory safety procedures.
   - (6) Manage PHSs in accordance with manufacturer recommendations regarding use, storage, and shelf-life considerations.
   - (7) Establish a "Designated Area," an area dedicated only to PHS work, when the PHS is present in an uncontained manner. The designated area may be the entire work area, a location in the work area, or a device, such as the laboratory chemical hood in the work area. Decontaminate PHS designated areas before using them for any other purpose.
     - (a) At a minimum, the immediate area surrounding the balance used for the weighing of TCs that are PHSs should be established as a "Designated Area."
     - (b) Label, tag, or mark PHS-designated areas to indicate the location's parameters, hazards, and requirements for each designated area.
     - (c) Decontaminate PHS designated areas after completing PHS work and before using them for any other purpose.
   - (8) Limit access to PHS designated areas as much as possible.
   - (9) Prohibit consuming food or beverages, using smoking products, chewing tobacco or gum, applying cosmetics or lip balm in PHS-designated areas.
   - (10) Ensure employees and associates who will work in designated areas and other areas where PHSs are stored or used have been informed and trained on the specific hazards, controls, and response procedures associated with such areas.

d. Personal Protective Equipment (PPE)
   (1) Obtain the appropriate PPE for the activity and understand how to put it on, use it, remove it, clean it, store it, and dispose of it properly. Read the product-specific SDS (Section 8) for recommended PPE.
   (2) Eye and Face Protection. At a minimum, use ANSI Z87.1-compliant safety glasses with affixed side shields for all PHS work. When your activity may produce splash, droplet, or spray hazards, use chemical splash goggles with indirect ventilation that are stamped with the ANSI Z87.1"D3" mark.
   (3) Hand Protection. Wear gloves resistant to the specific chemicals used in your activity. Wear gloves that are at least 6mil thick and 12 inches long; double-gloving and/or gloves at least 10mil thick should be worn for acute toxins, respiratory sensitizers, skin sensitizers, or specific target organ toxicity-single exposure (when the target organ effect may incapacitate exposed personnel). For the EASA procedures, chemicals will be handled as solids, liquids, and solutions (*e.g.*, solids or liquids dissolved in solvents). For the current chemicals involved in the EASA protocol: butyl gloves are recommended for working with or solutions containing acetone, acetonitrile, ethyl acrylate, and methyl pyruvate; and latex gloves are also acceptable. Nitrile gloves are recommended for working with all other solids, and neoprene gloves are recommended for use with nitric acid. If liquids come into contact with the gloves, the gloves should be removed and replaced. A composite (butyl/nitrile) or double-gloving (butyl, nitrile) may be employed for all EASA work. Additionally, a tri-polymer glove (*e.g.*, MAPA Trionic E-194), may be employed. However, MAPA Trionic gloves are comprised of natural rubber/neoprene/nitrile; accordingly, users may have an allergic response to the natural rubber component and the breakthrough time for acetone and acetonitrile is < 10 minutes. Therefore, the gloves must be changed immediately, whenever such liquids contact the gloves.
   (4) Skin and Body Protection. Wear personal clothing to cover potentially exposed skin fully (*i.e.*, long-sleeved shirt and long pants); use clothing that will not readily dissolve or react with the PHS. At a minimum, wear a laboratory coat or similar body protection for all PHS work. When the PHS work involves a large splash/droplet/spray hazard or acute toxins, wear a chemical-resistant laboratory coat, apron, or coveralls; wear arm gauntlets to protect arms, unless 18-inch gloves are worn. DO NOT leave arms and legs unprotected.
   (5) Foot Protection. Wear shoes closed in the heel and toe. When the PHS is highly hazardous, or a large splash/droplet/spray hazard exists, wear chemical-resistant overshoes or boots.

e. Safety/Emergency Equipment and Procedures
   (1) Emergency Eyewash Stations and Emergency Showers. Ensure an operating emergency eyewash station and emergency shower are in the work area where PHSs are stored or used. Confirm that the eyewash station and emergency shower are immediately accessible, no more than a 10 s walk from the chemical hazard, located in a well-lit area on the same level as the chemical hazard, and identified with a highly visible sign.
   (2) Spill Response Kits. Maintain spill control supplies (*e.g.*, absorbents, neutralizers) and appropriate PPE wherever PHSs are stored or used. Read the product-specific SDS (Section 6) for precise accidental-release measures and recommended spill control supplies. Spill-control supplies should be sufficient to control anticipated spill volumes.

5. Storage of Chemicals

a. Store in accordance with manufacturer recommendations. All chemicals except for methyl pyruvate, N-ethylmaleimide, and NBT can be stored at room temperature, while those three chemicals are stored at 4 C.
b. Minimize stored container sizes and quantities as much as possible.

c. Store in original, manufacturer-provided containers, whenever possible.
d. Store in tightly sealed, chemically resistant containers in secondary containment that is chemically resistant and large enough to contain any spills or releases.
e. Segregate from incompatible chemicals.

6. Procedures

a. General Safety Work Practices
   (1) Be prepared. Read the product-specific SDS (Section 4-6), and know the product-specific exposure, fire, and release/spill response procedures for your PHSs.
   (2) Know the location and proper use of the available safety/emergency response equipment (*e.g.*, fume hood, eyewash, safety shower, spill kit, fire extinguisher, fire alarm pulls). Make sure the path to emergency equipment is unobstructed.
   (3) Confirm that the required emergency eyewash station and safety shower are present, operational. Make sure the path to all required emergency equipment are unobstructed.
   (4) Confirm that any required, specialized exposure or spill response equipment is present, not expired, and immediately available.
   (5) Check PPE for any damage, put it on if it is not damaged, and wear it at all times when handling PHSs.
   (6) Confirm laboratory chemical hood is functioning properly before starting PHS work inside of the chemical hood.
   (7) Remove non-essential equipment from the immediate area where the PHS work will be conducted.
   (8) Ensure all containers, surfaces, and items in the immediate area where the PHS work will be conducted are compatible with the PHS.
   (9) Handle only the amount of PHS necessary to perform the activity; keep other containers and excess quantities in storage.
   (10) Perform all PHS work in secondary containment that is chemically resistant and large enough to contain any spills or releases, when possible to do so.
   (11) Open PHS containers carefully and slowly because the containers may be pressurized or release PHS gases, mists, or vapors when opening.
   (12) Dispense PHS manually or by gravity. DO NOT use air pressure as a means for dispensing.
   (13) Dispense PHS slowly in a manner that minimizes generation of splashes, droplets, and mists and prevents exposure to living tissue, metals, or other surfaces that are not compatible with PHS.
   (14) After pouring the required amount of PHS, wipe any drips off of the outside of the bottle of any drips, dry with a wipe, and place the bottle back in storage. Used wipes must be disposed of in a designated chemical waste container.
   (15) Avoid contacting and inhaling PHS during work.
   (16) Assume that containers, equipment, and surfaces inside an PHS Designated Area are contaminated with PHS. Never touch such containers, equipment, and surfaces without required PPE.
   (17) Immediately respond to all suspected and observed PHS exposures.
   (18) Immediately respond to all chemical exposures, releases, and spills.
   (19) Label any unattended PHS containers with chemical identity, its hazards, and your name. If it isn't feasible to label an unattended container, leave the container in the designated area, and place a sign in the immediate area indicating the chemical identity, its hazards, and your name as the chemical owner.
   (20) It may be necessary to conduct the NBT experiments in modified laboratory lighting conditions (*e.g.*, having some of the lights in the room turned off or adjusting the lighting so that only a fraction of the light spectrum is used) to limit photodegradation of the NBT reagent. If the lighting conditions differ from those typically used in the laboratory, it is important to ensure that there is sufficient lighting to perform all laboratory steps safely.

b. Safety Instructions for performing the assays:
   (1) Preparation of Solvent System and stock solutions
       Perform these processes in a fume hood.
   (2) Preparation of PC Solutions
       Conduct the weighing in the Nanoenclosure or in another space the limits the potential for inhalation. Add acetonitrile in a fume hood.
   (3) Preparation of TC Solutions (TC-S)
       Open the test chemicals first in a fume hood to observe whether there is any pressure in the container and visually evaluate the test substance. Conduct the weighing in either the Nanoenclosure or the fume hood, and mix the TC and solvent in the fume hood. For TCs that are liquid, perform the appropriate dilutions in the fume hood so that there is a 1 mM solution in dry acetonitrile.
c. Decontamination and Disposal
   (1) Wear all required PPE while performing PHS decontamination procedures.
   (2) Avoid contacting and inhaling PHS during cleanup.
   (3) Decontaminate exterior surfaces of containers and equipment potentially contaminated with PHS before removing them from the PHS Designated Area. Follow the decontamination procedures described in the product-specific SDS (Section 5 and 6).
   (4) Decontaminate the PHS Designated Area surfaces (*e.g.*, laboratory chemical hood bench top, faucet, faucet knobs, walls) before using them for any other purpose. Use paper towels to clean up the balances in the Nanoenclosure after each use and dispose of the paper towels as hazardous waste.
       (a) Follow decontamination procedures described in the product-specific SDS (Section 5 and 6).
       (b) Collect and treat all materials used for and collected from the decontamination procedures performed inside the PHS Designated Area as hazardous waste following applicable workplace-specific procedures.
   (5) Containers
       (a) Ensure decontaminated containers are properly labeled, tightly sealed, and returned to storage.
   (6) Waste Disposal
       (a) Contain, label, and turn in for disposal all spent, expired, or otherwise "waste" chemicals, such as used spill absorbents or contaminated articles, in accordance with the requirements of the responsible site environmental organization at the specific workplace.
       (b) To remove the liquid from the reservoirs or 96 well plates, use a vacuum aspirator. Periodically, pour the liquid from the waste flask for the vacuum aspirator to the waste container.
   (7) Personal Hygiene
       (a) Remove PPE before leaving the work area.
           i. Ensure PPE that will be re-used has been decontaminated and stored properly in the work area.
           ii. Ensure disposable and potentially contaminated PPE has been treated as hazardous waste.
       (b) Thoroughly wash hands and arms with soap and water upon leaving the work area.

Supplemental Figures

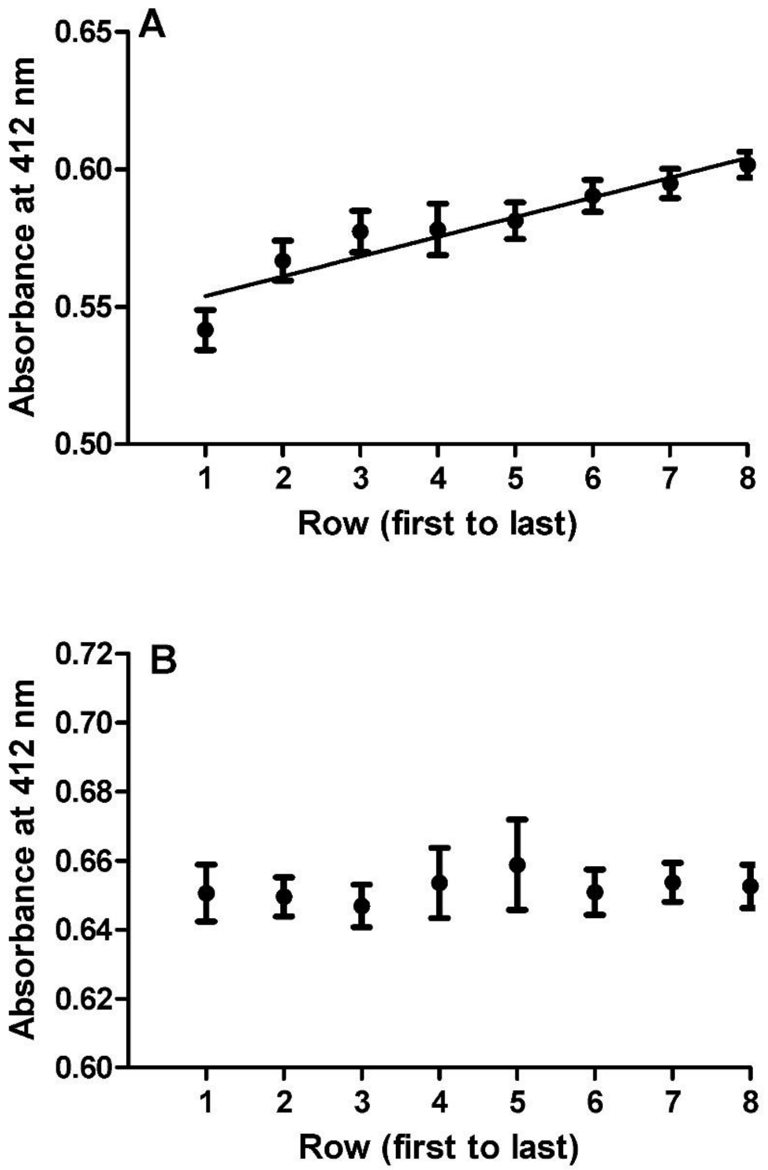

Figure S1. Photosensitivity: Absorbance of 4-nitrobenzenethiol (NBT) after loading in normal laboratory light (A) or under red light (B). Plates were loaded and then immediately placed in the plate reader. Absorbance of the NBT probe by row. Data points indicate mean values, and error bars indicate standard deviations of 12 replicates. The solid line is a linear regression fit of the data.

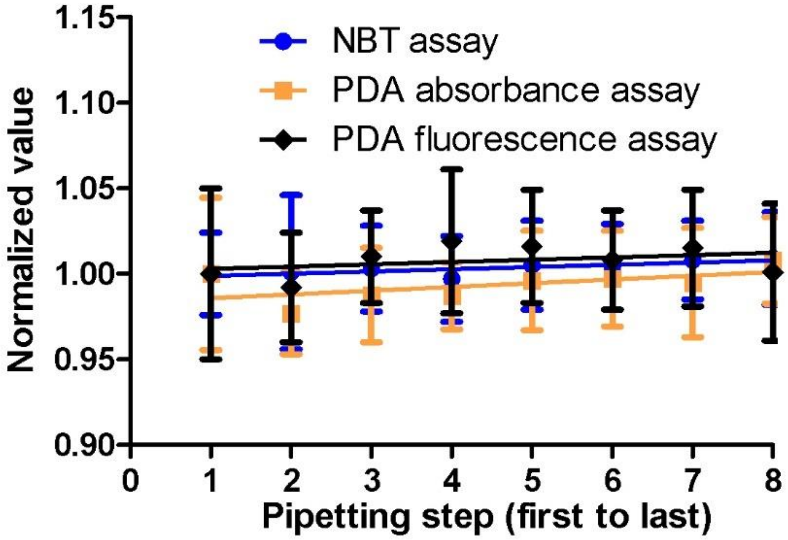

Figure S2. Pipetting Robustness Test: Between pipetting step comparison among each of the eight pipetting steps for all three assays for the negative control wells (n ≥ 13 plates). The mean absorbance or fluorescence values for the data for each pipetting step are normalized to those for the first pipetting step (column 2). All values are the mean and standard deviation values (n ≥ 104 wells for the first step and n ≥ 11 for the values in each of the other seven steps).

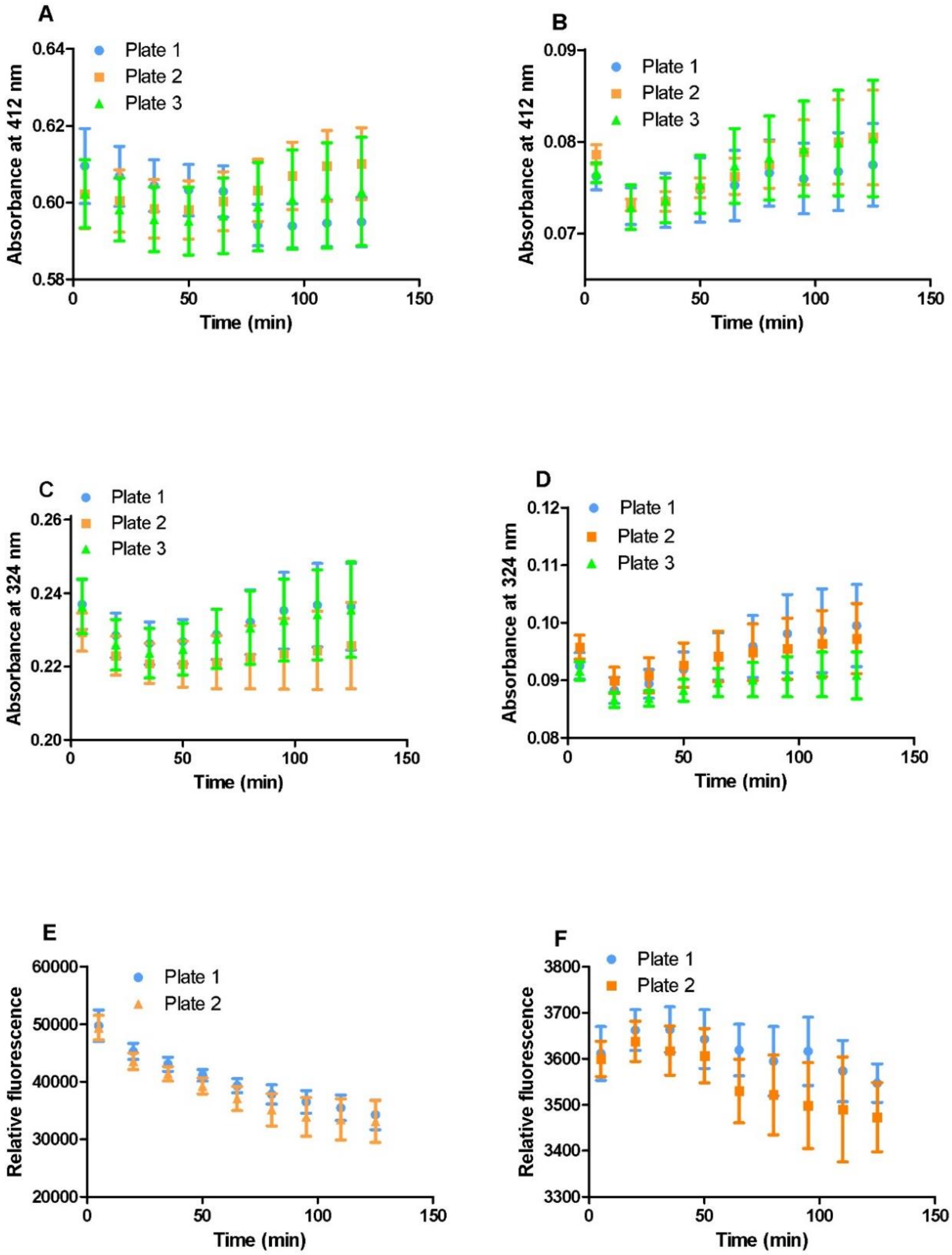

Figure S3. Negative Controls: Summary of results from negative control (4-nitrobenzenethiol (NBT) assay (A), pyridoxylamine (PDA) Absorbance (C), PDA Fluorescence (E)) and solvent system (NBT assay (B), PDA Absorbance (D), PDA Fluorescence (F)) during assay duration robustness testing. Data are the mean and error bars the standard deviation values for 15 or 8 values for the negative control and solvent system, respectively.

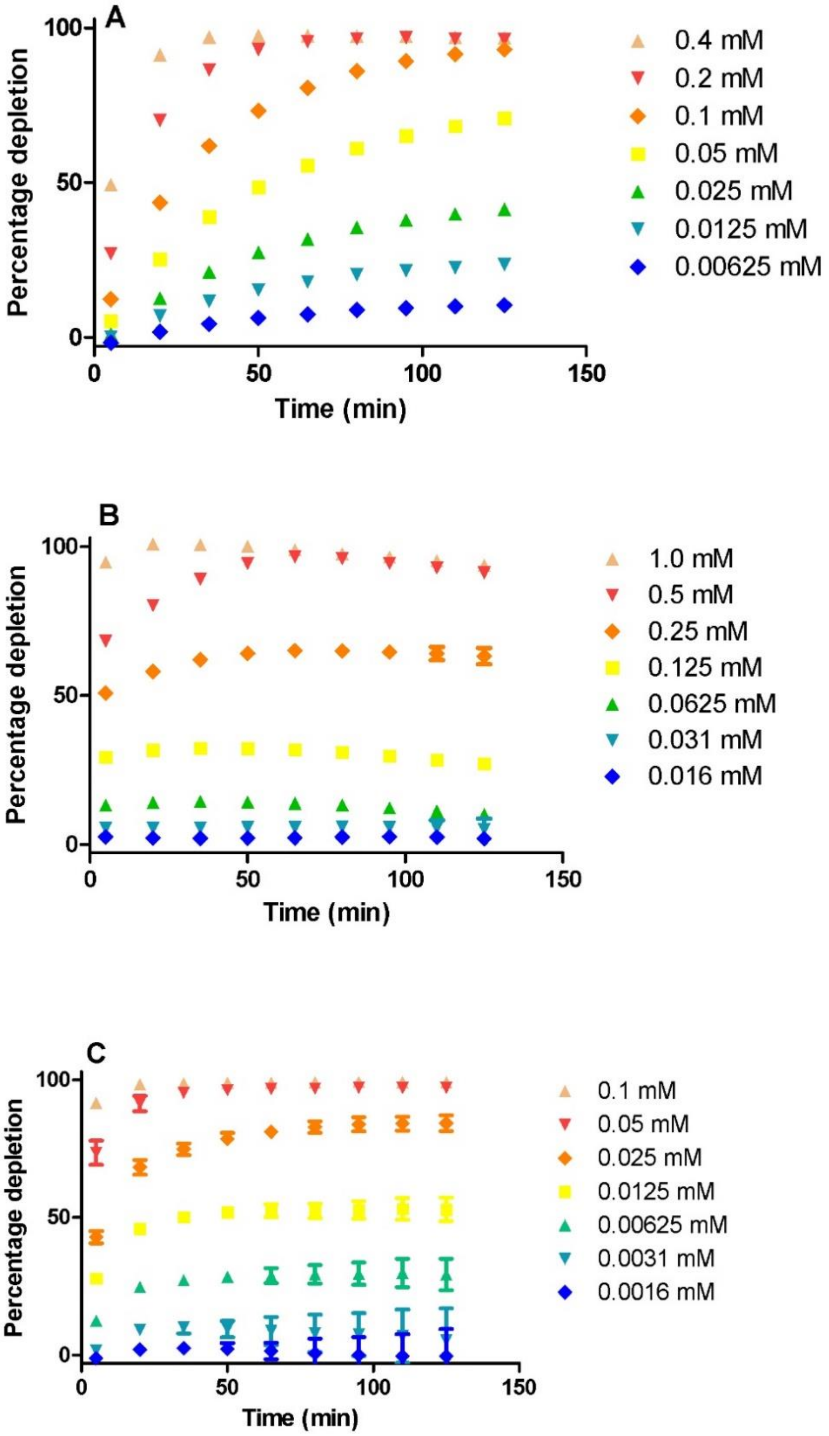

Figure S4. Positive Controls: Summary of results from testing the positive chemical control for the 4-nitrobenzenethiol (NBT) (A), pyridoxylamine (PDA) absorbance (B), and PDA fluorescence (C) assays for up to 2 h. Data indicate the mean and error bars the standard deviation values of three replicates. Error bars smaller than the data symbols are not visible.

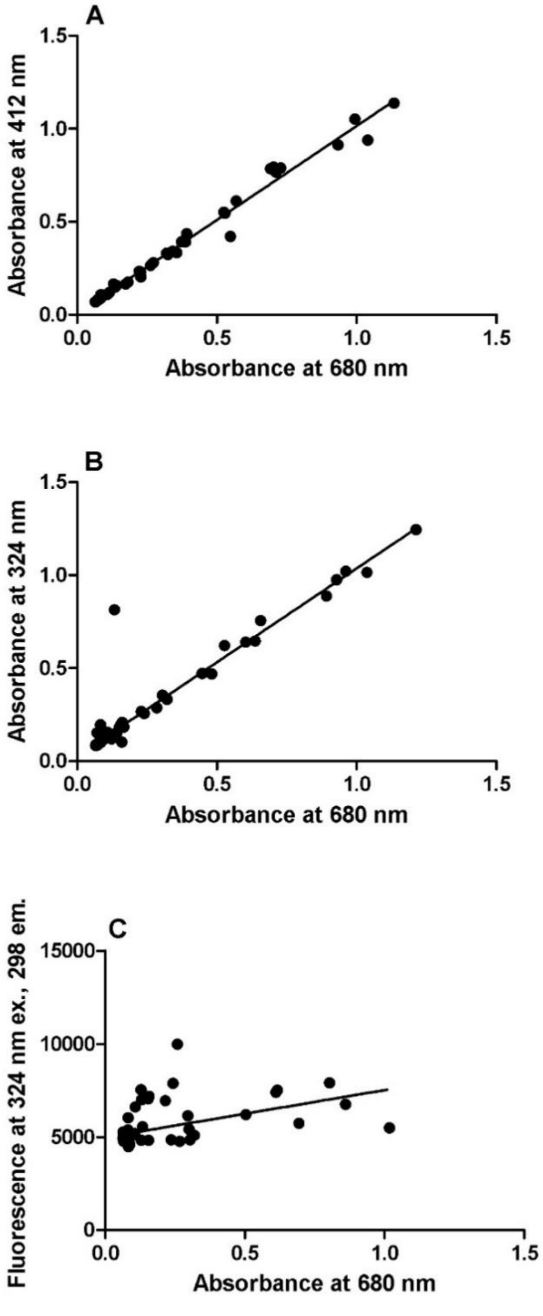

Figure S5. Bubble Test: Comparison of absorbance results at 412 nm (4-nitrobenzenethiol (NBT) assay) (A) or 324 nm (pyridoxylamine (PDA) absorbance assay) (B), or of fluorescence at 324 nm excitation and 298 emission (PDA fluorescence assay) (C) and 680 nm for a plate where bubbles were intentionally produced. Each data point reflects the data from an individual well. The solid line is a linear regression fit of the data.

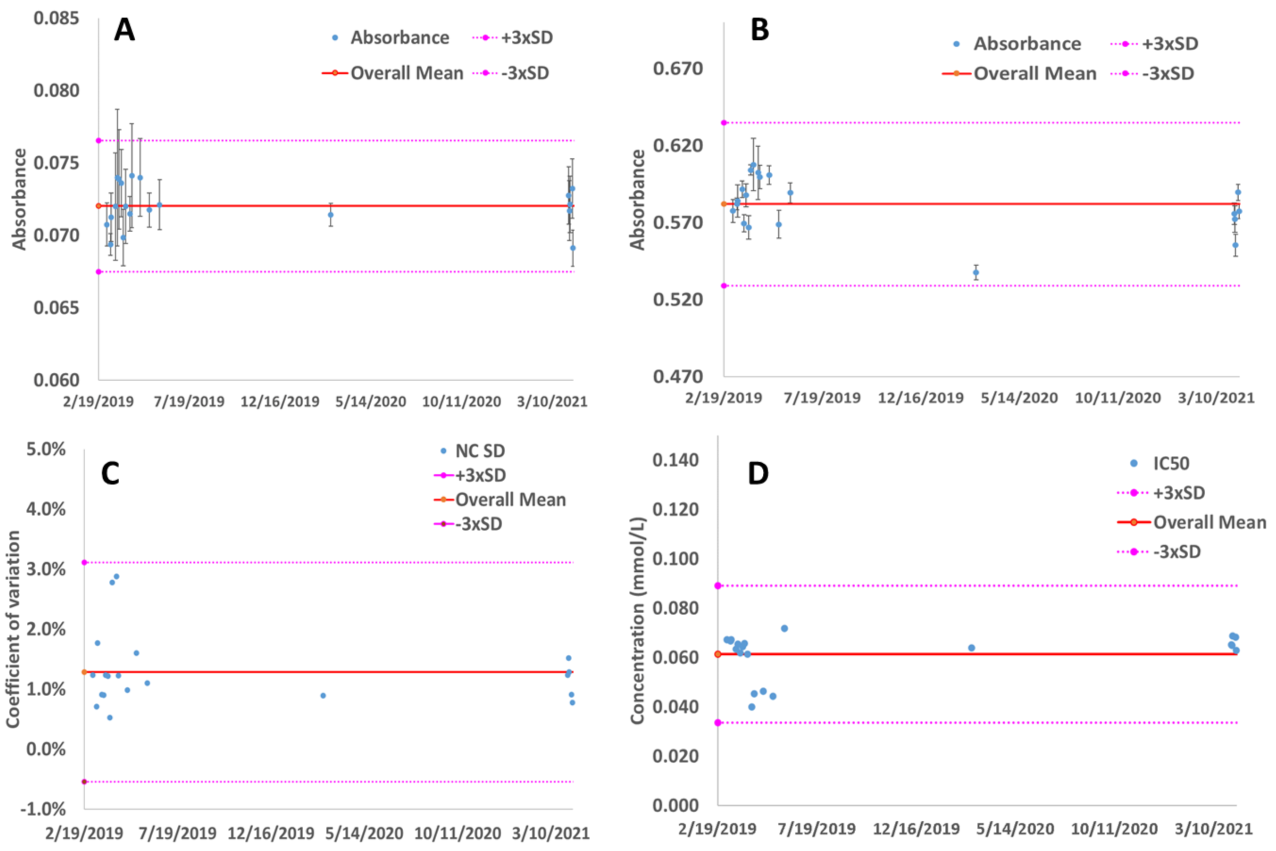

Figure S6. Control charting data for the NBT assay: solvent system (A), negative control (B), coefficient of variation (standard deviation/mean) for the negative control (C), and IC_{50} values of the positive control (D). Data indicate the mean and error bars indicate the standard deviation values of up to 15 replicates (A) or 8 replicates (B). The overall mean and standard deviation (SD) values were calculated based on the mean values across all time points. The mean and standard deviation values shown within part C are those of the coefficient of variation values.

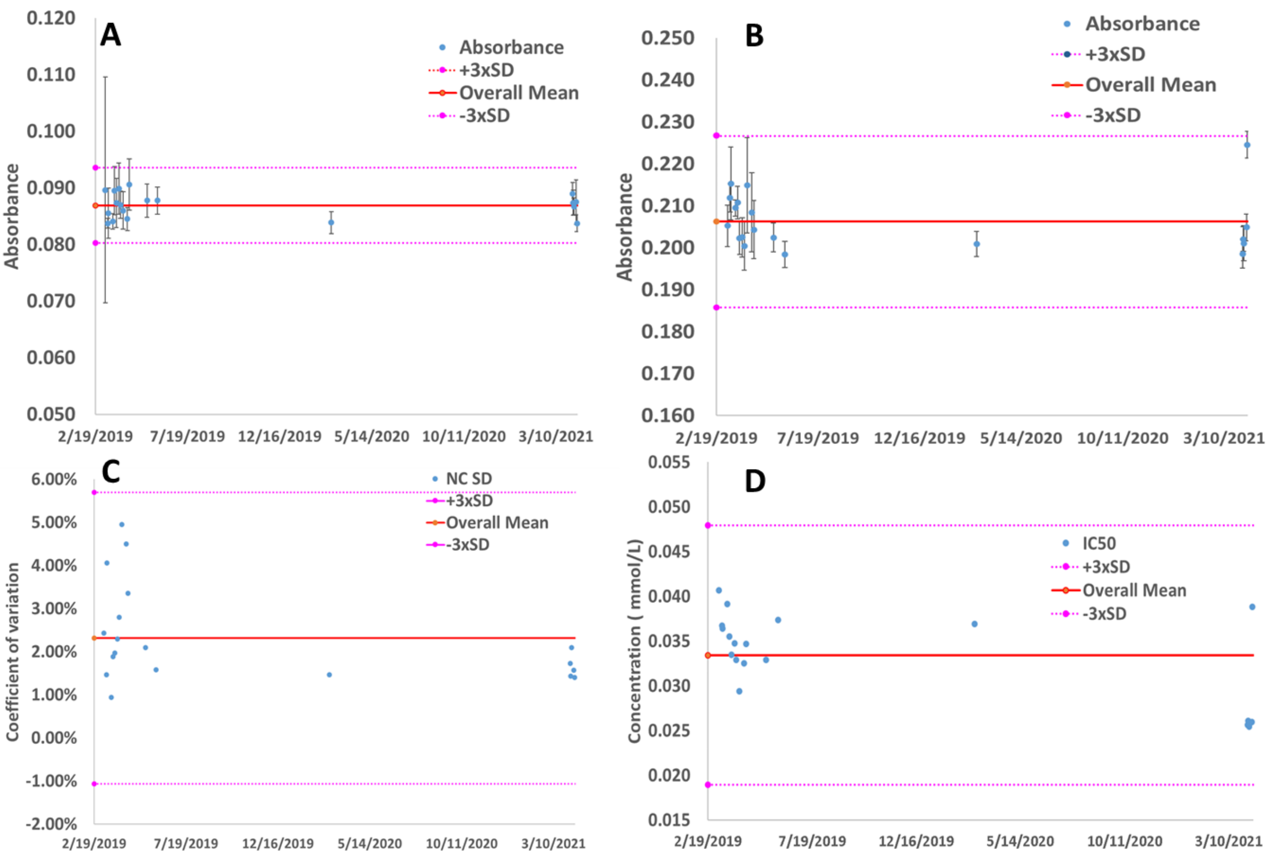

Figure S7. Control charting data for the PDA absorbance assay: solvent system (A), negative control (B), coefficient of variation (standard deviation/mean) for the negative control (C), and IC_{50} values of the positive control (D). Data indicate the mean and error bars indicate the standard deviation values of up to 15 replicates (A) or 8 replicates (B). The overall mean and standard deviation (SD) values were calculated based on the mean values across all time points. The mean and standard deviation values shown within part C are those of the coefficient of variation values.

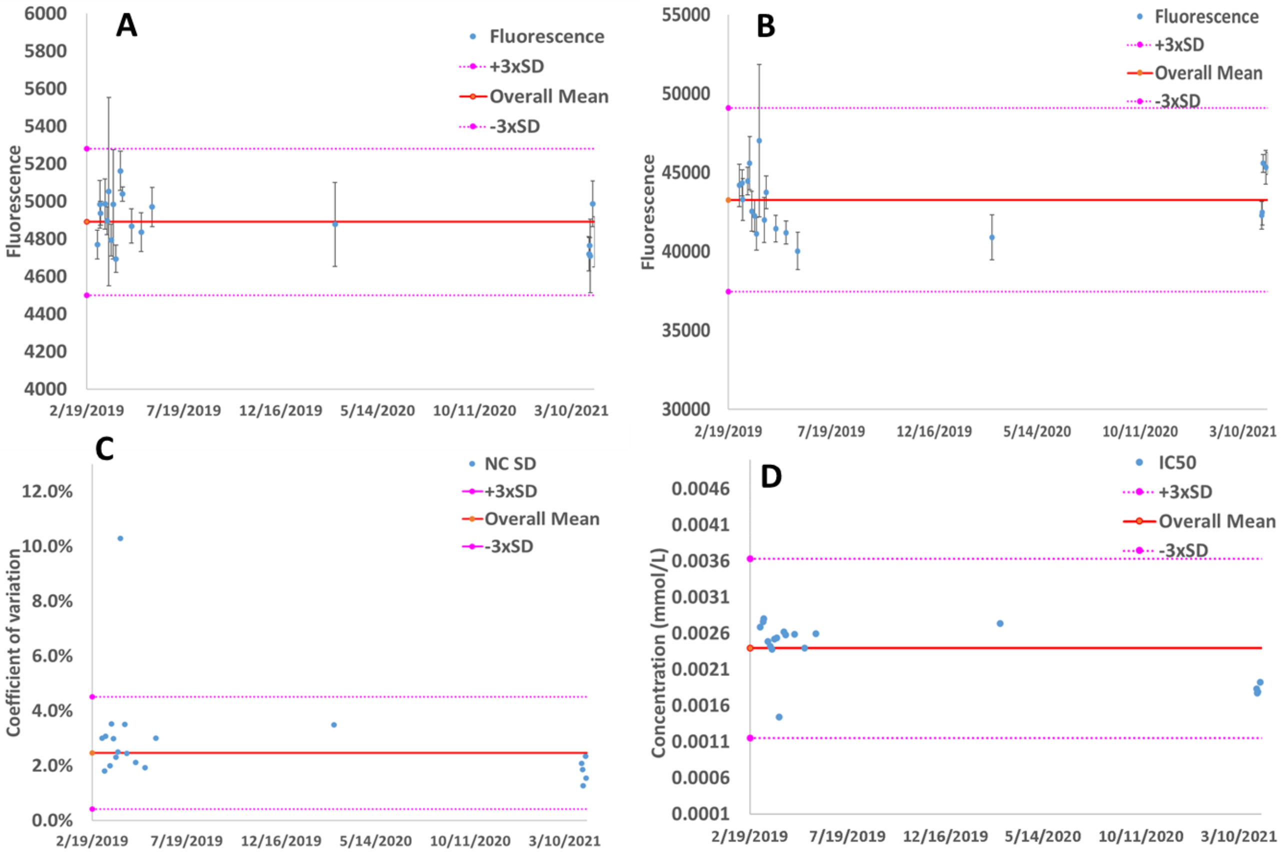

Figure S8. Control charting data for the PDA fluorescence assay: solvent system (A), negative control (B), coefficient of variation (standard deviation/mean) for the negative control (C), and IC$_{50}$ values of the positive control (D). Data indicate the mean and error bars indicate the standard deviation values of up to 15 replicates (A) or 8 replicates (B). The overall mean and standard deviation (SD) values were calculated based on the mean values across different dates. The mean and standard deviation values shown within part C are those of the coefficient of variation values.

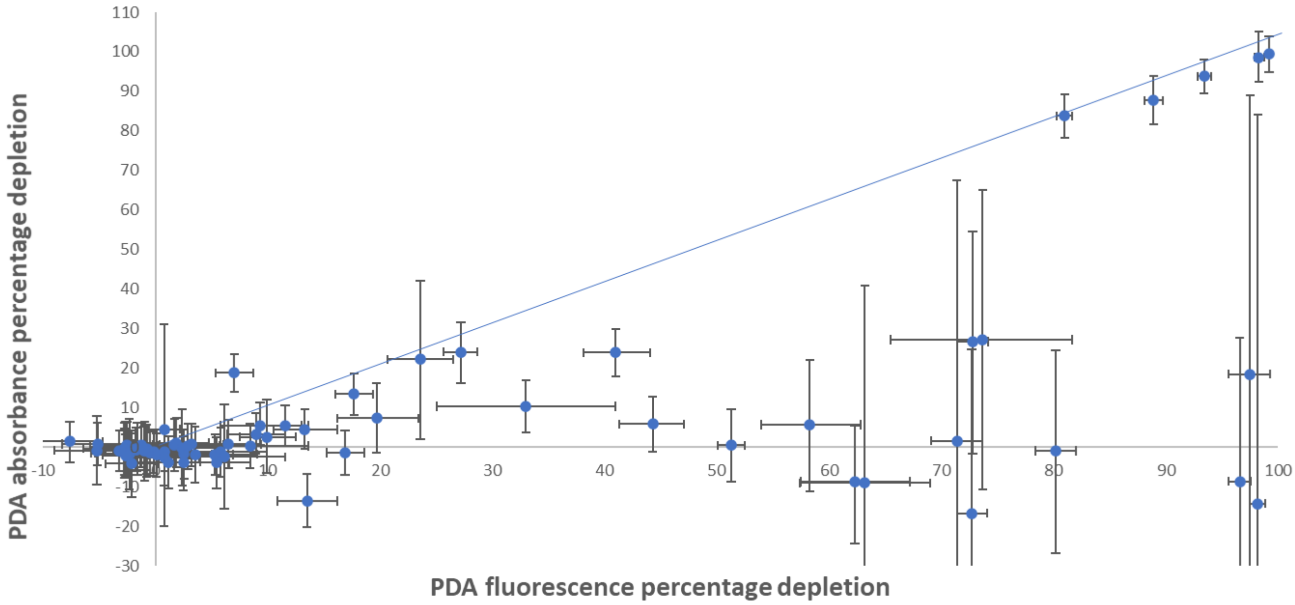

Figure S9. Comparison of values obtained from the PDA absorbance and PDA fluorescence percentage depletion for test compounds. Data points and error bars indicate the mean and 95 % confidence intervals determined using Bayesian modeling. Each data point represents the data for a TC in a particular run. Each data point shows both the PDA absorbance percentage depletion (y-axis) and the PDA fluorescence percentage depletion (x-axis) for an individual test chemical. The blue line indicates a 1:1 correlation line. The percentage depletion values were consistently higher for the PDA fluorescence assay and that the 95 % confidence intervals were also typically smaller for the fluorescence assay. None of the TCs showed a higher percentage depletion for the PDA absorbance assay as would be indicated by a data point above the blue line. Compounds with negative percentage depletion values less than -20 % or percentage depletion values greater than 110 % are excluded.
